# Necroptosis‐Mediated Synergistic Photodynamic and Glutamine‐Metabolic Therapy Enabled by a Biomimetic Targeting Nanosystem for Cholangiocarcinoma

**DOI:** 10.1002/advs.202309203

**Published:** 2024-06-05

**Authors:** Qichang Zheng, Tianhao Zou, Weimin Wang, Chen Zhang, Shaobo Hu, Xiang Cheng, Ran Liu, Guoliang Wang, Ping Sun, Xing Zhou, Bing Yang, Jianjun Xu, Yang Gao, Jinyang Gu

**Affiliations:** ^1^ Center for Liver Transplantation Union Hospital Tongji Medical College Huazhong University of Science and Technology Wuhan 430022 China; ^2^ Cancer Center Union Hospital Tongji Medical College Huazhong University of Science and Technology Wuhan 430022 China; ^3^ Department of Hepatobiliary Surgery Union Hospital Tongji Medical College Huazhong University of Science and Technology Wuhan 430022 China; ^4^ Key Laboratory of Organ Transplantation Ministry of Education; NHC Key Laboratory of Organ Transplantation; Key Laboratory of Organ Transplantation Chinese Academy of Medical Sciences Wuhan Hubei 430022 China

**Keywords:** cancer immunotherapy, cholangiocarcinoma, diselenide‐bond bridged mesoporous organosilica nanoparticles, glutamine‐metabolic therapy, necroptosis, tumor‐associated macrophages

## Abstract

Targeted delivery of glutamine metabolism inhibitors holds promise for cholangiocarcinoma therapy, yet effective delivery vehicles remain a challenge. This study reports the development of a biomimetic nanosystem, termed R‐CM@MSN@BC, integrating mesoporous organosilicon nanoparticles with reactive oxygen species‐responsive diselenide bonds for controlled release of the glutamine metabolism inhibitor bis‐2‐(5‐phenylacetamido‐1,3,4‐thiadiazol‐2‐yl) ethyl sulfide (BPTES) and the photosensitizer Ce6. Erythrocyte membrane coating, engineered with Arg‐Gly‐Asp (RGD) peptides, not only enhanced biocompatibility but also improved tumor targeting and tissue penetration. Upon laser irradiation, R‐CM@MSN@BC executed both photodynamic and glutamine‐metabolic therapies, inducing necroptosis in tumor cells and triggering significant immunogenic cell death. Time‐of‐flight mass cytometry analysis revealed that R‐CM@MSN@BC can remodel the immunosuppressive tumor microenvironment by polarizing M1‐type macrophages, reducing infiltration of M2‐type and CX3CR1^+^ macrophages, and decreasing T cell exhaustion, thereby increasing the effectiveness of anti‐programmed cell death ligand 1 immunotherapy. This strategy proposed in this study presents a viable and promising approach for the treatment of cholangiocarcinoma.

## Graphical abstract

The enhancement of solid tumor immunogenicity and resolution of the apoptosis resistance dilemma in tumors can be effectively achieved through the induction of necroptosis in tumor cells. R‐CM@MSN@BC demonstrates the capability of actively delivering photosensitizers and glutamine metabolism inhibitors, thereby inducing necroptosis in tumor cells and concurrently remodeling the immunosuppressive microenvironment.

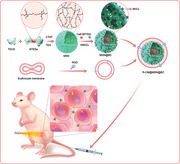

## 1 Introduction

Cholangiocarcinoma (CCA), arising from the epithelial cells of the bile duct, presents a formidable challenge with significant heterogeneity.^[^ ^1^ ^]^ The prognosis for advanced CCA is bleak, with an average overall survival ranging from 5–9 months due to the dearth of effective treatment modalities.^[^ ^2^, ^3^ ^]^ Immune checkpoint inhibitors (ICIs) that specifically target programmed cell death protein 1 (PD‐1)/programmed death‐ligand 1 (PD‐L1) have exhibited substantial clinical efficacy across various cancers.^[^ ^4^, ^5^, ^6^ ^]^ However, this therapeutic effect is observed in less than 20% of patients with advanced CCA.^[^ ^7^, ^8^ ^]^ The immunosuppressive tumor microenvironment (TME) plays a pivotal role in hampering the success of ICIs in patients with CCA by impeding cytotoxic T lymphocyte infiltration and activation.^[^ ^9^, ^10^ ^]^ Photodynamic therapy (PDT) has emerged as a promising intervention for CCA due to its tunable light‐induced damage, non‐invasiveness, and minimal side effects.^[^ ^11^, ^12^ ^]^ During PDT, photosensitizers generate cytotoxic reactive oxygen species (ROS) upon light irradiation, potentially inducing immunogenic cell death (ICD) and enhancing tumor cell immunogenicity.^[^ ^13^ ^]^ However, the rapid elimination of PDT‐generated ROS by elevated glutathione (GSH) levels within tumor cells diminishes the effectiveness of ICD, necessitating additional therapeutic strategies to potentiate PDT and reshape the immunosuppressive TME.

Tumor metabolism, particularly the reprogramming of glutamine utilization, significantly influences the immunosuppressive TME during tumor development.^[^ ^14^, ^15^, ^16^ ^]^ Tumor cells, in addition to heightened glucose demands, exhibit a dependency on glutamine, termed “glutamine addiction”.^[^ ^17^, ^18^, ^19^ ^]^ This metabolic trait poses challenges to immune cell function and undermines the success of cancer immunotherapy.^[^ ^20^, ^21^ ^]^ Inhibition of glutamine metabolism, specifically targeting key metabolic enzyme glutaminase 1 (GLS1), has been shown to remodel the TME by inducing M1‐type macrophage polarization and increasing cytotoxic T‐lymphocyte (CTL) infiltration in CCA cells.^[^ ^18^ ^]^ Glutamine metabolism acts as a metabolic checkpoint that inhibits immune cell‐mediated antitumor responses.^[^ ^11^, ^22^ ^]^ Furthermore, glutamine metabolism also significantly influences the regulation of intracellular redox homeostasis. The inhibition of glutamine metabolism in tumor tissues can lead to a reduction in the production of vital reducing agents, such as GSH and NADPH.^[^ ^23^, ^24^ ^]^ Thus, glutamine metabolism blockade could enhance treatment approaches that rely on oxidative damage, such as PDT, through a synergistic effect. The use of GLS1 inhibitors to block glutamine metabolism can synergistically improve PDT efficacy and alleviate the immunosuppressive TME. However, existing inhibitors targeting glutamine metabolism exhibit inadequate tumor tissue penetration, resulting in limited therapeutic effectiveness and substantial gastrointestinal adverse effects.^[^ ^25^ ^]^ Therefore, the development of a robust delivery system rooted in glutamine metabolism, with the ability to selectively target the CCA TME, holds significant promise for advancing therapeutic outcomes.

**Scheme 1:**
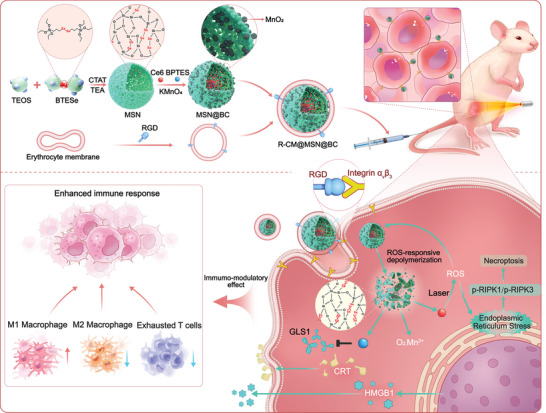
Schematic Illustration of the Synthesis Process and Therapeutic Mechanism of R‐CM@MSN@BC Nanosystem. R‐CM@MSN@BC is composed of a diselenide‐bond‐bridged hollow mesoporous organosilica core that exhibits responsiveness to reactive oxygen species (ROS). Moreover, the biomimetic nanosystem is enveloped by an erythrocyte membrane that has been subjected to modifications involving RGD peptides. This modification facilitates the selective targeting of α_v_β_3_ integrin, thereby facilitating the accurate administration and controlled release of nanomedicines. The diselenide‐bond bridged hollow mesoporous organosilica core is loaded with the GLS1 inhibitor BPTES and the photosensitizer Ce6, enabling the combination of glutamine metabolism therapy and photodynamic therapy (PDT). During the treatment process, R‐CM@MSN@BC can induce cell death in tumor cells through the necroptosis pathway, leading to the release of damage‐associated molecular patterns (DAMPs) that enhance the immunogenicity of cholangiocarcinoma. The combination of glutamine metabolism therapy and PDT holds promise in reshaping the immunosuppressive tumor microenvironment through reducing immunosuppressive macrophages, specifically M2‐type and CX3CR1^+^ macrophages, while concurrently promoting the infiltration of M1‐type macrophages. Consequently, this approach has the potential to enhance the efficacy of cholangiocarcinoma immunotherapy.

In this study, we engineered a biomimetic multifunctional controlled‐release drug nanosystem, designated as R‐CM@MSN@BC, to synergistically integrate PDT, glutamine metabolism therapy, and biological imaging. Illustrated in **Scheme** **1**, the nanosystem employed ROS‐responsive diselenide‐bond bridged hollow mesoporous organosilica nanoparticles (MSNs) as the framework, loaded with the photosensitizer Ce6 and the GLS1 inhibitor BPTES. Upon light irradiation, PDT‐induced ROS cleaved the diselenide bond, causing MSN skeleton disintegration and complete release of Ce6 and BPTES. The outer surface of the nanosystem was coated with erythrocyte membrane modified with Arg‐Gly‐Asp (RGD) peptide, which enhanced biocompatibility, targeting, and penetration capabilities toward 3D tumor spheroids via specific interaction between RGD peptide and overexpressed integrin α_ν_β_3_ on CCA cell surfaces. Experimental findings confirmed significant ROS production by R‐CM@MSN@BC during therapeutic intervention, enhancing CCA immunogenicity through necroptosis induction in tumor cells. Importantly, time‐of‐flight mass cytometry (CyTOF) data indicated R‐CM@MSN@BC‐mediated glutamine metabolism inhibition, which reduced immunosuppressive macrophages—particularly M2‐type and CX3CR1^+^ macrophages—and augmented M1‐type macrophage infiltration. Furthermore, the treatment decreased exhausted T cell infiltration, reversing the immunosuppressive CCA TME. When combined with immune checkpoint blockade therapy, R‐CM@MSN@BC significantly inhibited both primary and abscopal tumor growth. Thus, our results highlight the safety and efficacy of the “Trinity” biomimetic nanosystem as a comprehensive strategy for CCA treatment.

## 2 Results and Discussion

### 2.1 GLS1 as a Potential Therapeutic Target in CCA

**Figure 1:**
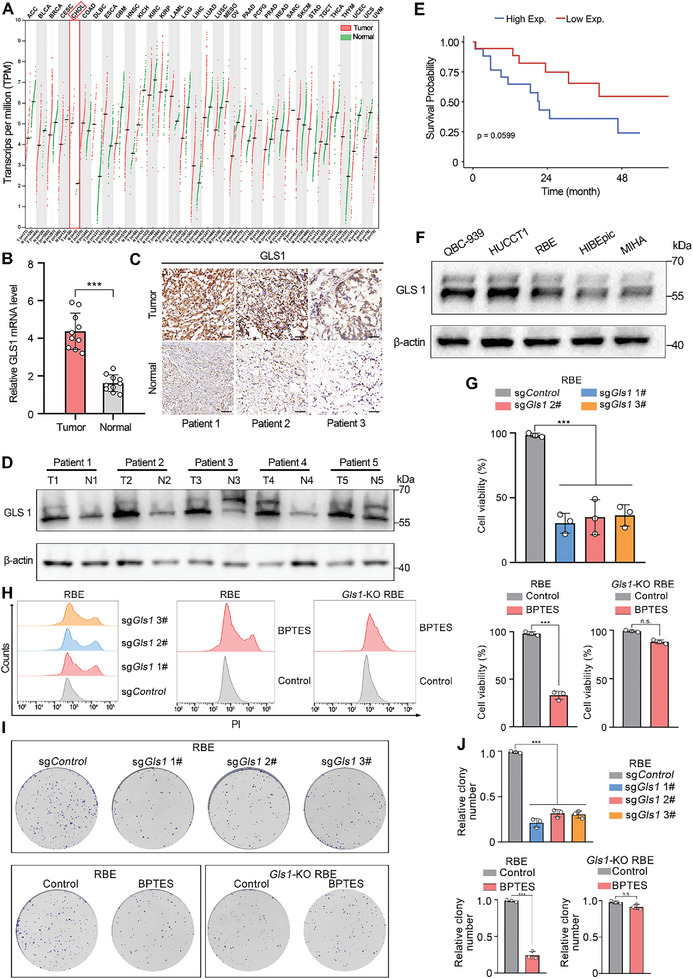
GLS1 was abundantly expressed in cholangiocarcinoma patients' tumor tissues and associated with a poor prognosis. A) Pan‐cancer analysis of GLS1 expression across cancers from TCGA and GTEx datasets. Abbreviations: BLCA: Bladder urothelial carcinoma; BRCA: Breast invasive carcinoma; CHOL: Cholangiocarcinoma; COAD: Colon adenocarcinoma; ESCA: Esophageal carcinoma; HNSC: Head and neck squamous cell carcinoma; KICH: Kidney chromophobe; KIRC: Kidney renal clear cell carcinoma; KIRP: Kidney renal papillary cell carcinoma; LIHC: Liver hepatocellular carcinoma; LUAD: Lung adenocarcinoma; LUSC: Lung squamous cell carcinoma; PRAD: Prostate adenocarcinoma; READ: Rectum adenocarcinoma; STAD: Stomach adenocarcinoma; THCA: Thyroid carcinoma; UCEC: Uterine corpus endometrial carcinoma. B) Relative mRNA expression levels of GLS1 in 10 pairs of CCA and matched non‐tumor tissues from our institution; n = 10 per group. C) GLS1 expression in CCA tissues were determined using the immunohistochemistry (IHC) staining. Scale bar, 100 µm. D) Western blot analysis of GLS1 expression in CCA and matched non‐tumor tissues. N: normal liver tissue, T: tumor tissue; n = 3 per group. E) High expression of GLS1 in CCA patients predicts a poor prognosis based on TCGA dataset. F) Western blot and its quantification analysis of GLS1 expression in total cell extracts from human cholangiocarcinoma cells and human normal cells. G) The viability of RBE cells or GLS1‐KO RBE cells after different treatment. H) Flow cytometry analysis of dead cells in RBE cells or GLS1‐KO RBE cells after different treatments. I, J) Representative images and its corresponding quantitative analysis of the RBE cells or GLS1‐KO RBE cells after different treatment, assessed with colony‐forming assay. n = 3 per group n.s., no significance; *P < 0.05; **P < 0.01; ***P < 0.001.

Given the essential role of the glutamine metabolic pathway in tumor cell proliferation and the significance of GLS1 within this pathway, we conducted a comprehensive analysis across 33 tumor types from the The Cancer Genome Atlas (TCGA) database to evaluate GLS1 mRNA expression. Our analysis revealed a substantial increase in GLS1 expression across various tumors compared to corresponding normal tissues. Particularly noteworthy was the significantly elevated GLS1 expression in CCA tissues compared to normal tissues (**Figure** **1**A; Figure S1A, Supporting Information). Confirmation of GLS1 overexpression in CCA tissues was achieved through real‐time quantitative reverse transcription polymerase chain reaction, western blot, and immunofluorescence staining in a set of 10 paired samples (Figure 1B–D; Figure S1B, Supporting Information). Prognostic analysis of 36 CCA cases from the TCGA database indicated that elevated GLS1 expression in tumor tissues correlated with a poorer clinical prognosis (Figure 1E). Subsequent assessment of GLS1 protein levels in CCA cell lines (QBC‐939, RBE, HuCCT1) and human normal tissue cell lines (HIBEpic, MIHA) using western blot analysis demonstrated a significant upregulation of GLS1 protein levels in tumor cell lines compared to normal tissue cell lines (Figure 1F; Figure S1C, Supporting Information). These findings provide strong evidence for the high expression of GLS1 in CCA and its association with an unfavorable patient prognosis. In order to ascertain the efficacy of GLS1 inhibition in suppressing CCA cell growth and proliferation, we utilized CRISPR/Cas9 technology to knock down the GLS1 gene in QBC‐939 and RBE cell lines, resulting in the generation of GLS1‐KO CCA cells (Figure S1D, E, Supporting Information). Subsequently, the alterations in cell activity and proliferative potential of CCA were investigated following the inhibition of GLS1. The experimental findings indicating that either GLS1 gene knockdown or treatment with the GLS1 inhibitor BPTES significantly impeded the cell activity and proliferative potential of CCA cells. However, this effect was unaffected after the use of BPTES on GLS1‐KO CCA cells (Figure 1G–J; Figure S1F–H, Supporting Information). Thus, the above experimental data suggest that BPTES‐mediated GLS1 suppression can inhibit the growth and proliferation of CCA cells. The suppression of glutamine metabolism leads to decreased production of reducing agents within the TME, thereby potentially improving the effectiveness of PDT. Consequently, the combination of glutamine metabolism therapy targeting GLS1 with PDT holds promise as a viable treatment approach for CCA.

### 2.2 Fabrication, Characterization, and ROS‐Responsive Degradation of R‐CM@MSN@BC

**Figure 2:**
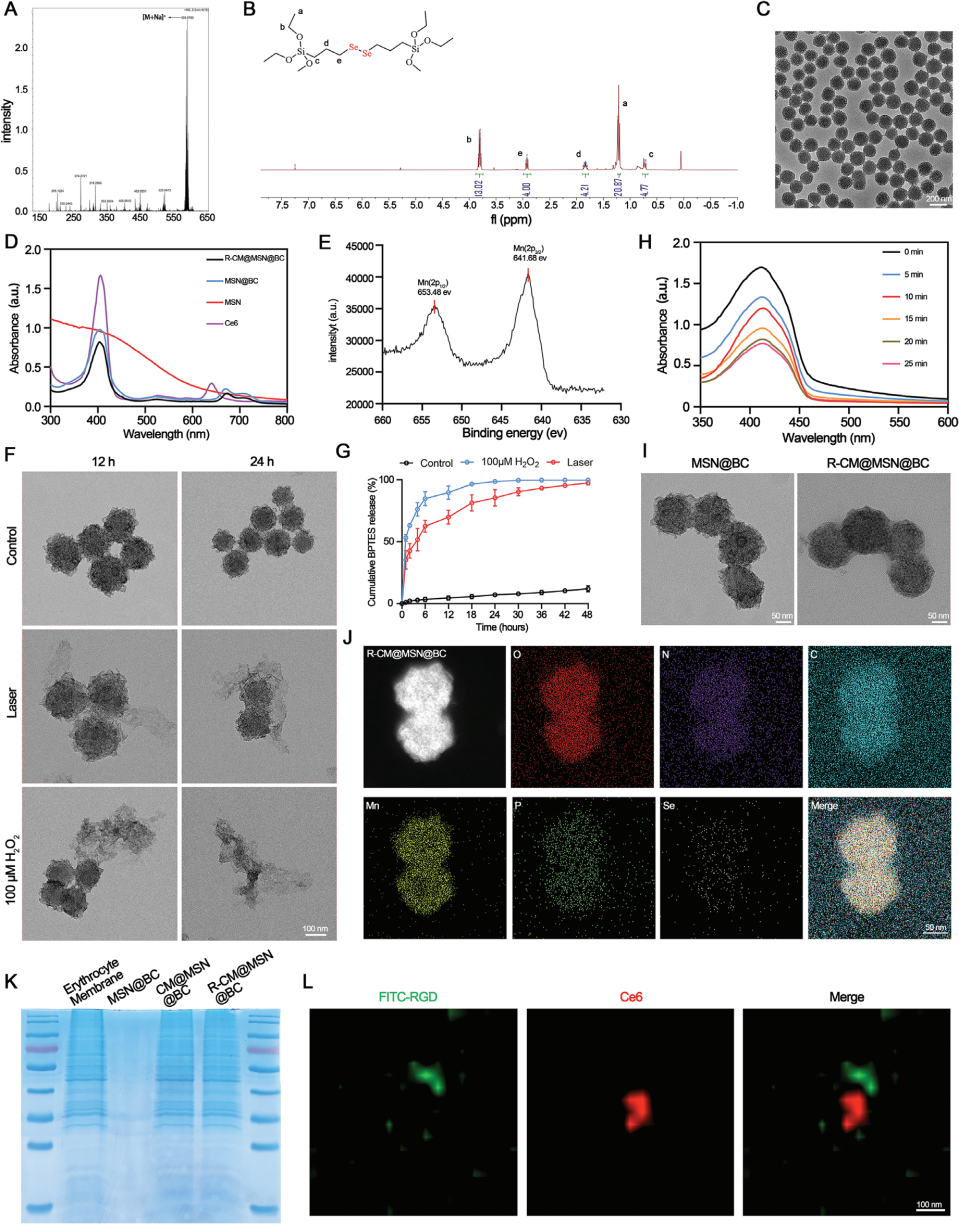
Fabrication, Characterization and ROS‐responsive degradation of R‐CM@MSN@BC. A, B) Mass spectrometry and ^1^H NMR spectra of BTESe. C) Representative TEM images of MSN. Scale bar, 200 nm. D) UV–vis‐NIR spectra of Ce6, MSN, MSN@BC, R‐CM@MSN@BC. E) XPS spectra for Mn 2p. F) Representative TEM images of MSN@BC after 660 nm laser irradiation (0.3 W cm^−2^, 3 min) or incubation in H_2_O_2_ (100 µM) at 12 h or 24 h. Scale bar, 100 nm. G) The cumulative BPTES‐release profile of MSN@BC in H_2_O_2_ (100 µM) or 660 nm laser irradiation (0.3 W cm^−2^, 3 min). H) The absorption spectra of DPBF treated with MSN@BC upon different times of laser irradiation (660 nm, 0.2 W cm^−2^). I) Representative TEM images of R‐CM@MSN@BC. Scale bar, 50 nm. J) Representative element‐mapping images of R‐CM@MSN@BC. Scale bar, 50 nm. K) SDS‐PAGE analysis of empty erythrocyte membrane, MSN@BC, CM@MSN@BC and R‐CM@MSN@BC. L) Representative spinning disk confocal super resolution microscope images of R‐CM@MSN@BC. Scale bar, 100 nm.

The drug release kinetics of mesoporous silica are influenced by the dimensions of its pores, resulting in the retention of certain drugs within the material and hindering rapid and complete drug release. Additionally, the slow degradation of the mesoporous silica framework raises concerns about its safety in vivo, thereby restricting its clinical utility. Consequently, there is a pressing necessity to enhance traditional mesoporous silica nanomaterials with regulated drug delivery capabilities. A hollow mesoporous silica bridged by diselenide bond (MSN) was synthesized using ethyltrimethylammonium tosylate and triethanolamine as catalysts. The diselenide containing organosilica precursor bis(3‐(triethoxysilyl)propyl) diselenide (BTESe) and inorganic silica precursor tetraethyl orthosilicate were employed in the synthesis process (Figure S2A,B, Supporting Information). Prior to synthesis, BTESe was prepared and characterized through mass spectrometry and ^1^H nuclear magnetic resonance spectra (**Figure** **2**A,B). Transmission electron microscopy (TEM) images revealed the spherical and hollow structure of MSN (Figure 2C). The Brunauer–Emmet–Teller surface area, total pore volume, and average pore size of the MSNs were determined to be 461.73 m^2^ g^−1^, 1.081 cm^3^ g^−1^, and 9.36 nm, respectively (Figure S2C,D, Supporting Information). The photosensitizer Ce6 and GLS1 inhibitor BPTES were loaded into the pores of the thin shell and hollow structure using negative pressure adsorption, resulting in successful loading into the MSN. Ce6 loading was confirmed by UV‐Vis‐NIR and fluorescence spectra of various nanoplatforms. A slight redshift observed at 654 nm to 673 nm may be attributed to hydrophobic interactions and π–π stacking between the aggregated Ce6 in the core (Figure 2D). Loading content was determined by analyzing the supernatant Ce6 and BPTES using high‐performance liquid chromatography. The maximum loading content of Ce6 was approximately 7.32%, and BPTES reached 6.96%. Subsequently, MSN was briefly mixed with manganese permanganate (KMnO_4_) to enable the in situ formation of MnO_2_. As indicated in Figure 2E, the chemical state of Mn was investigated. Characteristic peaks at 653.48 and 641.68 eV corresponded to the Mn (IV) 2p_1/2_ and Mn (IV) 2p_3/2_, indicating the predominant existence of Mn^4+^ in the MnO_2_ of MSN@BC. To investigate the ROS‐responsive properties of MSN@BC, MSN@BC was immersed in phosphate‐buffered saline (PBS) and subjected to different treatments. TEM was used to observe morphological changes and structural evolution, revealing clear structural breakdown and collapse of the majority of MSN@BC particles in a PBS solution containing 100 µM H_2_O_2_ after a 24‐h incubation period. Furthermore, the backbone of MSN@BC experienced significant degradation when exposed to specific light wavelengths (Figure 2F). Notably, after 3 minutes of laser irradiation, MSN@BC demonstrated a drug release rate of 63.9% after 6 h and 73.6% after 12 h (Figure 2G). To confirm the production of singlet oxygen (^1^O_2_) by MSN@BC, the generation of ^1^O_2_ in MSN@BC following irradiation with a 655 nm laser was assessed by monitoring the absorbance of 1,3‐diphenylisobenzofuran at 416 nm. As shown in Figure 2H, the treated MSN@BC groups exhibited significantly reduced absorption, indicative of ^1^O_2_ generation. These results reveal that MSN@BC exhibits good ROS‐responsive degradation behavior.

To enhance the biocompatibility and tumor‐targeting capabilities of MSN@BC, we employed a method in which the erythrocyte membrane, modified with RGD peptide, was enveloped onto the surface of MSN@BC, resulting in R‐CM@MSN@BC synthesis. R‐CM@MSN@BC exhibited a core‐shell structure, with the MSN core encapsulated within a thin and smooth membrane shell. The membrane thickness of R‐CM@MSN@BC was ≈12 nm, similar to the thickness of the erythrocyte membrane (Figure 2I; Figure S2E, Supporting Information). Energy‐dispersive X‐ray spectroscopy mapping verified the presence and distribution of Si, Mn, Se, and P within a single R‐CM@MSN@BC entity, indicating uniform wrapping of the erythrocyte membrane around R‐CM@MSN@BC (Figure 2J). Subsequently, the nanosystems were subjected to sodium dodecyl sulfate‐polyacrylamide gel electrophoresis experiments. The results revealed that both R‐CM@MSN@BC and CM@MSN@BC exhibited an identical protein composition to that of erythrocyte membranes, confirming the successful encapsulation of erythrocyte membranes surrounding MSN (Figure 2K). In order to identify the presence of the RGD peptide modification on R‐CM@MSN@BC, the RGD peptide was labeled with a FITC probe and observed using a spinning disk confocal super‐resolution microscope. We observed that the FITC fluorescence enveloped the red fluorescence emitted by Ce6 in the nanosystem, further confirming the successful encapsulation of MSN@BC nanoparticles loaded with Ce6 by the erythrocyte membrane modified with FITC‐RGD peptide (Figure 2L). Fluorescence spectroscopy experiments provided additional evidence, as R‐CM@MSN@BC exhibited distinct fluorescence peaks at 525 nm and 660 nm, corresponding to FITC and Ce6 fluorescence peaks, respectively (Figure S2F, Supporting Information). The dynamic light scattering assay revealed that the diameter of R‐CM@MSN@BC measured 141.62 nm, indicating an increase of ≈13 nm compared to the MSN@BC particle size. This increase can be attributed to the successfully encapsulated erythrocyte membrane. Furthermore, the surface potential of the coated erythrocyte membrane with MSN@BC changed from −39.35 to −26.76 mV, further confirming the successful coating of erythrocyte membrane (Figure S2G,H, Supporting Information). The in vivo results showed that 24 hours after injection of the nanosystems and 5‐minute light stimulation, a decrease in GLS1 protein expression was detected in mouse tumor tissues, suggesting that R‐CM@MSN@BC also functions as a good ROS‐responsive degrader in vivo (Figure S2I, Supporting Information).

### 2.3 Biosafety and Imaging Performance of R‐CM@MSN@BC

**Figure 3:**
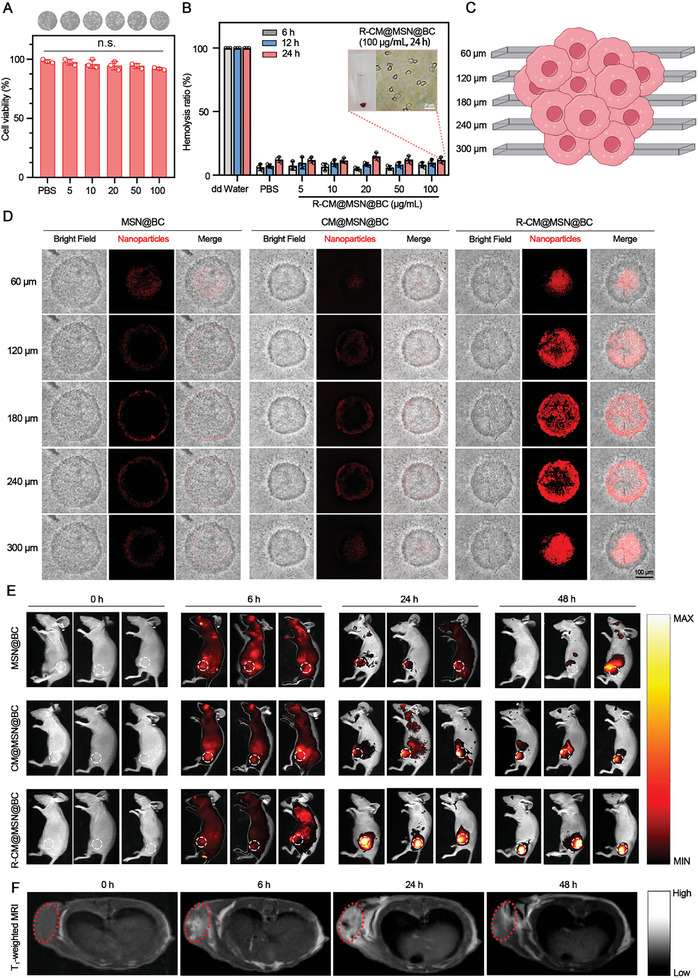
Biosafety and Imaging Performance of R‐CM@MSN@BC. A) The viability of HIBEpic cells after co‐incubation with R‐CM@MSN@BC (5, 10, 20, 50, 100 µg mL^−1^) for 24 h; n = 3 per group. B) Hemolysis ratio and representative images of erythrocyte in the presence of R‐CM@MSN@BC with various concentrations at 6 h, 12 h and 24 h, respectively; n = 3 per group. Scale bar, 20 µm. C) Schematic of fluorescence in various transverse sections with a depth interval of 60 µm of RBE cholangiocarcinoma cell spheroid. D) Bright‐field (BF) and fluorescence images of different transverse sections with an increasing depth of 60 µm of RBE cell spheroids cultured under various conditions. Scale bar, 100 µm. E) Fluorescent images of the RBE tumor‐bearing Balb/c nude mice at different time points after injection of R‐CM@MSN@BC (5 mg k^−1^g). F) T1‐weighted MRI of the RBE tumor‐bearing Balb/c nude mice at different time points after injection of R‐CM@MSN@BC (5 mg k^−1^g).

Effective biosafety and advanced visualization capabilities are essential requirements for the successful clinical implementation of diagnostic‐integrated nanosystems. The cytotoxicity of various nanosystems was evaluated in vitro using HIBEpic cells. Results from **Figure** **3**A and Figure S3A (Supporting Information) suggest that when coated with an erythrocyte membrane, CM@MSN@BC and R‐CM@MSN@BC displayed minimal toxicity toward HIBEpic cells. R‐CM@MSN@B was unable to generate a sufficient amount of reactive oxygen species to cleave the diselenide‐bond, as the photosensitizer Ce6 was not present within it (Figure S3B, Supporting Information). Consequently, the release of drugs contained within the nanoshells was impeded, thus limiting their cytotoxic effects. Additionally, the addition of R‐CM@MSN@BC to peripheral blood at a concentration of 100 µg mL^−1^ did not lead to significant hemolysis, indicating favorable biocompatibility (Figure 3B). To assess the targeting properties of the RGD peptide on the surface of the nanosystems for CCA cells, we conducted co‐incubation experiments with RBE cells and assessed uptake efficiency using confocal laser scanning microscopy and flow cytometry. R‐CM@MSN@BC exhibited superior tumor targeting capabilities and uptake efficiency than those exhibited by MSN@BC. Furthermore, this effect was observed to be attenuated in the presence of the integrin α_ν_β_3_ inhibitor cilengitide (Figure S3C–F, Supporting Information). The enhanced tumor targeting and uptake efficiency of R‐CM@MSN@BC facilitate selective eradication of tumor cells exposed to laser irradiation, enhancing light‐controlled inhibition efficacy and significantly mitigating adverse effects on non‐targeted cells and tissues (Figure S3G, Supporting Information). In the context of solid tumor therapy, drug effectiveness relies on permeability. We accordingly assessed the tumor penetration characteristics of the nanosystems using a 3D tumor spheroid model comprising RBE cells. Different nanosystems were co‐cultured with tumor spheroids for 24 h, followed by imaging using a confocal high‐intensity imaging system at a consistent depth interval of 60 µm (Figure 3C). The spheroids incubated with R‐CM@MSN@BC exhibited significantly stronger red fluorescence of Ce6 in each section than that exhibited by the groups cultured with MSN@BC and CM@MSN@BC (Figure 3D). This indicates that the R‐CM@MSN@BC nanosystem exhibits heightened penetration with the RGD modification, which effectively enhances the targeted binding of the nanosystem to RBE cells that exhibit α_v_β_3_ integrin overexpression on their cellular surfaces.

To scrutinize the dual imaging capability and biosafety of R‐CM@MSN@BC in vivo, we intravenously administered the nanosystems to mice bearing RBE tumors, followed by scanning using an in vivo fluorescence imaging system. The results highlight that the integration of RGD peptides into the erythrocyte membrane coating of R‐CM@MSN@BC amplifies its tumor targeting ability and extends its circulation time in the body. Consequently, a significant quantity of fluorescent signals persisted in the tumor tissues of mice 48 hours post‐injection of R‐CM@MSN@BC (Figure 3E, Figure S3J,K, Supporting Information). The MnO_2_ on the surface of MSN can undergo reactions with hydrogen peroxide and hydrogen ions within the TME, resulting in the production of manganese ion (Mn^2+^). Mn^2+^ can reduce the relaxation time of tumor tissue during the T1 phase of magnetic resonance imaging (MRI), thereby boosting the sensitivity of MRI for both tumor diagnosis and treatment evaluation. We investigated the potential of R‐CM@MSN@BC to function as an MRI enhancer. As depicted in Figure 3F and Figure S2L (Supporting Information), the enhanced T1 phase post‐administration of R‐CM@MSN@BC displayed improved tumor visibility compared to pre‐enhancement imaging. Furthermore, the assessment of vital organ functions in various groups of mice after 3 days of treatment revealed no notable abnormalities in the groups treated with nanoparticles, as demonstrated by both biochemical indicator tests (Figure S3H, Supporting Information) and histological examination (Figure S3I, Supporting Information). These results affirm that the application of R‐CM@MSN@BC, involving the modification of erythrocyte membranes with RGD peptide, brings about improvements in blood circulation, tumor targeting, permeation, and tumor imaging capabilities.

### 2.4 Potent ROS Generation and Antitumor Efficiency of R‐CM@MSN@BC

**Figure 4:**
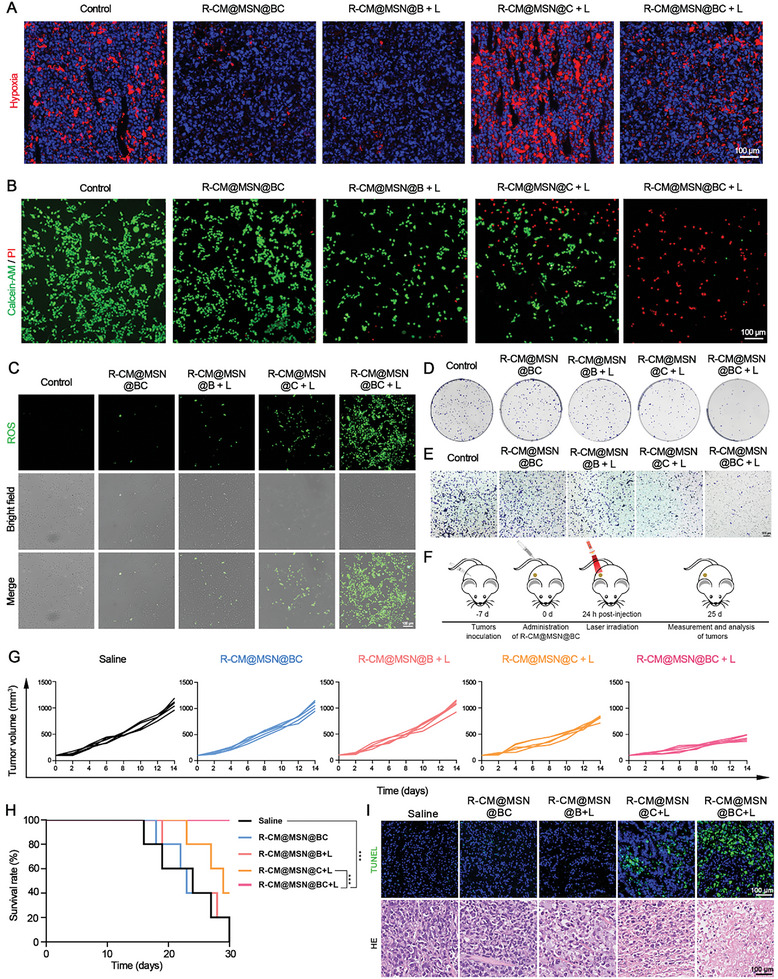
Robust ROS Production and Antitumor Effect of R‐CM@MSN@BC. A) Representative hypoxia of RBE cells of tumor slices after different treatments. Balb/c mice bearing RBE tumors were i.v. injection with indicated nanoparticles. Scale bar, 100 µm. B) Fluorescence microscope images of RBE cells treated with the different NPs. Cells were stained with Calcein‐AM and propidium iodide. Scale bar, 100 µm. C) Representative ROS fluorescence images of RBE cells s after different treatments. Scale bar, 100 µm. D, E) Representative images of the RBE cells after exposure to the indicated nanosystems, assessed with colony‐forming assay (D) and transwell assays (E). F) Schematic diagram of in vivo anti‐tumor experiments on RBE tumor‐bearing nude mice. G) Individual tumor growth kinetics of mice receiving the indicated treatments. H) Survival rates of RBE tumor‐bearing nude mice that experienced above different treatments; n = 5 per group. I) H&E and tunel staining images of tumor slices harvested from RBE tumor‐bearing nude mice after different treatments. Scale bar, 100 µm. *P < 0.05; **P < 0.01; ***P < 0.001.

Building upon these promising experimental findings, we conducted additional assessments of ROS production and the antitumor efficiency of R‐CM@MSN@BC. As the MnO_2_ in the nanosystem reacts with H_2_O_2_ as well as H^+^ in the tumor tissue to generate oxygen. Therefore, we tested the effect of different nanosystems on the amelioration of the hypoxic microenvironment in tumor tissues. We conducted western blot analysis to assess hypoxia‐inducible factor (HIF)−1α levels in treated RBE cells after different treatment. Notably, there was a significant reduction in HIF‐1α levels in cells treated with CM@MSN@BC and R‐CM@MSN@BC groups compared to the control group, providing additional evidence of hypoxia alleviation (Figure S4A,B, Supporting Information). Subsequently, various nanosystems were administered via the tail vein to RBE tumor‐bearing Balb/C nude mice, followed by tumor tissue staining using a hypoxic probe (Hypoxyprobe‐1 plus kit) after 24 h. The tumor tissues of mice injected with R‐CM@MSN@BC nanosystems exhibited a notable reduction in hypoxia in comparison to the control group, primarily due to the oxygen‐generating capabilities of R‐CM@MSN@BC (**Figure** **4**A). Since PDT is an oxygen‐consuming process, injection R‐CM@MSN@C significantly exacerbated the degree of hypoxia in the tumor tissues of the mice after laser irradiation. Interestingly, group R‐CM@MSN@BC + L significantly mitigated tumor tissue hypoxia compared to group R‐CM@MSN@C + L (Figure 4A; Figure S4C, Supporting Information). We hypothesized that this was attributed to the synergistic effects of BPTES and Ce6, resulting in enhanced tumor cell death and reduced oxygen consumption in tumor tissues following light exposure. Consequently, we conducted investigations into the suppressive impact of R‐CM@MSN@BC on glutamine metabolism and its capacity to product ROS. After conducting metabolite analysis on mouse tumor tissue, the inclusion of BPTES in R‐CM@MSN@BC was observed to significantly inhibit tumor glutamine metabolism and the associated production of reductants (Figure S4E, Supporting Information). Moreover, after laser irradiation, the R‐CM@MSN@BC group exhibited a significantly higher production of ROS than the R‐CM@MSN@C group. This result can be attributed partially to the inhibitory effect of the responsively released BPTES in R‐CM@MSN@BC on the production of reducing substances such as GSH, leading to a decrease in ROS consumption. On the other hand, the presence of MnO_2_ in R‐CM@MSN@BC, which supplies an ample quantity of O_2_ for PDT to generate ROS (Figure 4C, Figure S4D, Supporting Information). The results of live/dead cell staining experiments demonstrated that R‐CM@MSN@BC exhibited enhanced cytotoxicity toward tumor cells within the same treatment duration (Figure 4B). The in vitro therapeutic efficacy of R‐CM@MSN@BC was evaluated against RBE cells. Due to the ability to efficiently generate ROS, R‐CM@MSN@BC demonstrates high efficacy in inducing cell death in cholangiocarcinoma cells and the therapeutic efficiency of R‐CM@MSN@BC was irradiance and light irradiation time‐dependent (Figure S4F–H, Supporting Information). Treatment of R‐CM@MSN@BC led to significant impairments in cloning formation and migration abilities following laser treatment, demonstrating the most pronounced cell‐killing effect (Figure 4D,E).

We further investigated the in vivo antitumor effects of R‐CM@MSN@BC. Subcutaneous RBE tumors were induced in immunocompromised mice, and the nanosystems were intravenously administered via the tail vein 7 days following tumor inoculation. Light was applied to the tumor site after 24 hours (Figure 4F). By day 14, the average tumor mass of mice in groups R‐CM@MSN@B, R‐CM@MSN@C and R‐CM@MSN@BC was measured at 1.18 g, 0.88 g, and 0.42 g, respectively. Utilizing the CDI formula,^[^ ^26^ ^]^ the CDI of the R‐CM@MSN@BC group was calculated to be 0.404, suggesting a significant synergistic effect between the glutamine metabolism inhibitor BPTES and the photosensitizer Ce6 in the biomimetic nanosystem. At the same time, the R‐CM@MSN@BC group exhibited a substantial tumor suppression rate of 63.06%, whereas the R‐CM@MSN@C group exhibited a tumor suppression rate of only 26.32% (Figure 4G, Figure S4I–K, Supporting Information). The survival rate of the remaining five tumor‐bearing mice was assessed, and after 30 days of continuous monitoring, the R‐CM@MSN@BC + L group exhibited a survival rate of 100% (Figure 4H). Tunel and hematoxylin and eosin (H&E) staining revealed a significant reduction in cell proliferation within the tumor tissue of the R‐CM@MSN@BC + L group (Figure 4I). These results were attributed to the synergistic effects of glutamine inhibition and photodynamic therapy, resulting in tumor cell death and achieving a “1+1>2” effect.

### 2.5 R‐CM@MSN@BC Induces Tumor Cell Death through the Necroptosis Pathway

**Figure 5:**
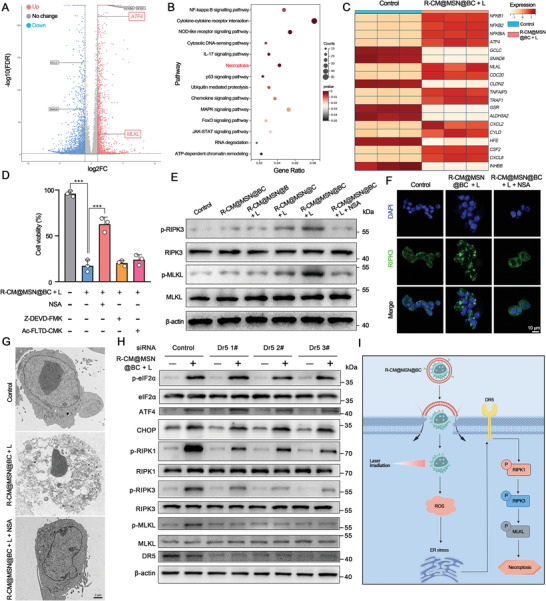
R‐CM@MSN@BC Mediates Tumor Cell Death Through the Necroptosis Pathway. A) The transcriptome of the RBE of Control and R‐CM@MSN@BC plus laser. The screen hits were selected with cut‐offs set at fold change > 2 and P‐value < 0.05. Red, upregulated. Blue, downregulated. B) Signaling pathway enrichment analysis of Kyoto Encyclopedia of Genes and Genomes (KEGG) in R‐CM@MSN@BC plus laser–treated RBE cells. C) The top 20 up‐ and down‐regulated genes in RBE cells treates with R‐CM@MSN@BC plus laser. D) Effect of cell‐death‐suppressing compounds on R‐CM@MSN@BC plus laser. MLKL inhibitor NSA (10 µM), apoptosis inhibitor Z‐DEVD‐FMK (20 µM), pyroptosis inhibitor Ac‐FLTD‐CMK (5 µM); n = 3 per group. E) Western blot images of necroptosis‐related proteins after different treatments. The levels of indicated proteins were determined by immunoblotting. n = 3 independent biological repeats. F) The effects of R‐CM@MSN@BC plus laser on the formation of RIP3 punctation, the distribution of RIPK3 (green) and DAPI (blue) was detected using confocal microscopy. G) TEM analysis of RBE cells after different treatment. Scale bars, 1 µm. H) RBE cells were transfected (48 h) with control siRNA, or sequentially distinct DR5 siRNA, then treated with PBS and R‐CM@MSN@BC plus laser, respectively. The levels of indicated proteins were determined by immunoblotting. n = 3 independent biological repeats. I) A schematic model to illustrate the mechanism of necroptosis in tumor cells caused by R‐CM@MSN@BC plus laser (By Figdraw).

Nanomedicines typically impede tumor growth by inducing apoptosis in tumor cells.^[^ ^27^ ^]^ However, the development of resistance to apoptosis is a characteristic feature in the progression of malignant tumors.^[^ ^28^ ^]^ Necroptosis, a form of cell death known for eliciting more pronounced ICD effects than apoptosis, is initiated by substantial levels of ROS.^[^ ^29^, ^30^ ^]^ Building on previous experiments demonstrating that R‐CM@MSN@BC generates significant ROS quantities through the synergistic action of Ce6 and BPTES, we explored the potential of R‐CM@MSN@BC to induce necroptosis in CCA cells. To decipher the mechanism underlying R‐CM@MSN@BC‐induced tumor cell death, we conducted RNA sequencing (RNA‐seq) analysis on R‐CM@MSN@BC + laser‐treated RBE cells. Differential gene expression analysis was performed, identifying upregulated and downregulated genes with a 2‐fold change cutoff and a significance [adjusted P (P_adj_)] value of <0.05 (**Figure** **5**A). Kyoto Encyclopedia of Genes and Genomes (KEGG) functional analysis revealed enrichment in the necroptosis pathway in RBE cells treated with R‐CM@MSN@BC gene products, suggesting potential induction of necroptosis following laser irradiation (Figure 5B). To validate RNA‐seq findings at the protein level, we initially confirmed the expression of receptor‐interacting protein kinase 3 (RIPK3, a crucial initiator and effector of necroptosis) in CCA cells. Results revealed a high level of RIPK3 expression (Figure S5A, Supporting Information). Introducing various inhibitors targeting different death pathways to RBE cells treated with R‐CM@MSN@BC, our findings indicated that necrosulfonamide (NSA), a specific mixed lineage kinase domain‐like pseudokinase (MLKL) inhibitor, effectively suppressed cell death induced by R‐CM@MSN@BC (Figure 5D,E; Figure S5D, S6B,C, Supporting Information). Moreover, NSA prevented RIPK3 punctation and cell swelling, characteristic morphological changes associated with necroptosis caused by R‐CM@MSN@BC (Figure 5F,G; Figure S5B, Supporting Information). In vivo experiments demonstrated a notable elevation in the phosphorylation of RIPK3 in tumor tissues of mice subjected to treatment with R‐CM@MSN@BC, as evidenced by fluorescent staining (Figure S5I, Supporting Information). Moreover, necroptosis, a highly immunogenic form of cell death, resulted in the release of substantial quantities of damage‐associated molecular patterns. Membrane translocation of calreticulin and release of high mobility group box 1 were notably enhanced following treatment with R‐CM@MSN@BC in combination with laser (Figure S5G, H, Supporting Information). These results suggest that R‐CM@MSN@BC can trigger subsequent ICD effects by inducing necroptosis in tumor cells.

To elucidate the downstream pathways through which ROS generated by R‐CM@MSN@BC induce necroptosis in tumor cells, we conducted a comparative analysis of RNA‐seq results. This revealed a significant increase in the expression levels of marker genes associated with endoplasmic reticulum stress in RBE cells following treatment with R‐CM@MSN@BC (Figure 5C). Additionally, increased protein levels of activating transcription factor 4, C/EBP homologous protein, and phosphorylation of eukaryotic translation initiation factor 2 subunit 1 were consistently observed after treatment with R‐CM@MSN@BC in conjunction with laser therapy (Figure 5H; Figure S6, Supporting Information). Small interfering RNA (siRNA) was employed to investigate the involvement of various death receptors in R‐CM@MSN@BC‐mediated necroptosis. Findings indicate that death receptor 5 (DR5) deficiency, but not tumor necrosis factor receptor 1/2 (Tnfr1/2) and Fas (also called CD95), inhibits the phosphorylation of receptor‐interacting protein kinase (RIPK1), RIPK3, and MLKL, consequently suppressing necroptosis (Figure 5H; Figure S5E,F; Figure S6, Supporting Information). These results suggest that DR5 acts as an upstream regulator of necroptosis in the context of R‐CM@MSN@BC combined with laser treatment (Figure 5I).

### 2.6 R‐CM@MSN@BC Modulates the Immunosuppressive Microenvironment of CCA

**Figure 6:**
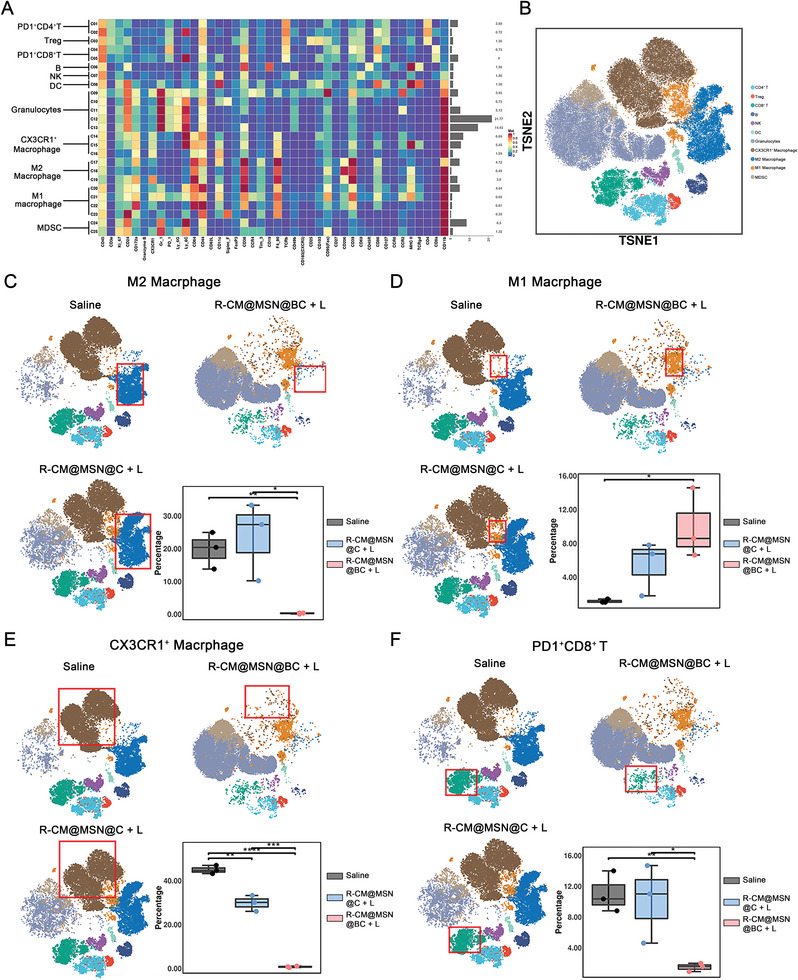
R‐CM@MSN@BC Reshapes the Immunosuppressive Microenvironment of CCA. A) Heatmap depicting median signal intensity (MSI) of indicated markers derived from CyTOF. B) A t‐distributed stochastic neighbor embedding (tSNE) plot via nonlinear dimensionality reduction showing the immune cell clusters identified by CyTOF analysis. C to F) T‐SNE plots showing expression levels of (C) M2‐type macrophage, (D) M1‐type macrophage, (E) CX3CR1^+^ macrophage and (F) PD1^+^CD8^+^ T cells with corresponding quantitative comparisons. n = 3 per group. n.s., no significance; *P < 0.05; **P < 0.01; ***P < 0.001.

To comprehensively examine the impact of R‐CM@MSN@BC on the TME of CCA, we established a subcutaneous transplanted CCA model in C57BL/6J mice with a normal immune response. The CCA tissue underwent treatment and was analyzed using CyTOF. Our metal‐labeled antibody panel was designed to identify primary immune cell populations infiltrating the tumor, including markers for cell lineage, function, and cytokine activity. CyTOF data revealed 11 distinct intratumoral immune cell populations, and the t‐distributed stochastic neighbor embedding (t‐SNE) algorithm was employed for data analysis (**Figure** **6**A,B). The immune landscape of tumors treated with R‐CM@MSN@BC plus laser (R‐CM@MSN@BC + L) significantly differed from both saline‐treated tumors and tumors treated with R‐CM@MSN@C plus laser (R‐CM@MSN@C + L), particularly in terms of the distribution of tumor‐associated macrophages (TAMs) and T cells (Figure S7A, Supporting Information). The R‐CM@MSN@BC + L group showed a significant reduction in the M2‐type macrophage subpopulation (CD206^+^ macrophage) and the inhibitory macrophage subpopulation (CX3CR1^+^ macrophage) compared to the saline or R‐CM@MSN@C + L group, indicating a decrease in immunosuppressive macrophages within TAMs (Figure 6C,E). Additionally, R‐CM@MSN@BC + L treatment increased the M1‐type macrophage subpopulation (CD86^+^ macrophage), suggesting enhanced proinflammatory effects (Figure 6D). Tumor‐infiltrating T cell exhaustion is correlated with tumor progression and immunotherapy resistance. In contrast to control groups receiving saline and R‐CM@MSN@C + L, CyTOF analysis revealed a decrease in PD‐1 expression among intratumoral CD8^+^ and CD4^+^ T cells in mice treated with R‐CM@MSN@BC + L (Figure 6F, Figure S7B,C, Supporting Information). These findings collectively indicate that R‐CM@MSN@BC + L effectively modifies the immunosuppressive TME of CCA by reducing immunosuppressive macrophages, specifically M2‐type macrophages and CX3CR1^+^ macrophages, and augmenting M1‐type macrophage infiltration. Furthermore, the treatment leads to a decrease in exhausted T cell infiltration, attributed to the inhibitory impact of BPTES on glutamine metabolism within R‐CM@MSN@BC.

### 2.7 Enhanced Systemic Immune Response with R‐CM@MSN@BC in Combination with Anti‐PD‐L1

**Figure 7:**
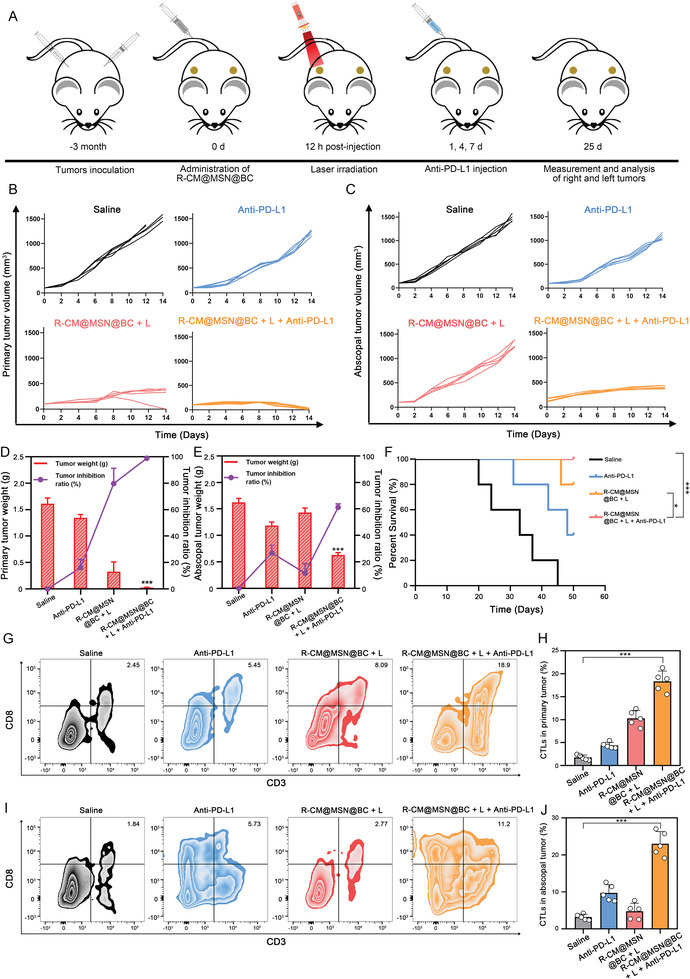
R‐CM@MSN@BC in Combination with Anti‐PD‐L1 Effectively Enhances Systemic Immune Response. A) Schematic diagram of different treatments for inhibiting primary and abscopal tumor on C57BL/6 mice subcutaneous graft tumor model. B, C) Individual primary and abscopal tumor growth kinetics of mice receiving the indicated treatments. D, E) Primary and abscopal tumor weight and inhibition ratio of various treated mice. F) Survival rates of C57BL/6J tumor‐bearing mice that experienced above different treatments (n = 5). G, H) CD3^+^CD8^+^ T cells proportions in primary tumor evaluated by flow cytometry and its quantitative analysis. I, J) CD3^+^CD4^+^ T cells proportions in primary tumor evaluated by flow cytometry and its quantitative analysis. n = 5 per group. n.s., no significance; *P < 0.05; **P < 0.01; ***P < 0.001.

Immunotherapy is a commonly utilized clinical strategy for the treatment of tumors. At present, Anti‐PD‐L1 are the recommended immunotherapeutic agents for CCA; however, their therapeutic effectiveness is limited due to the immunosuppressive TME of CCA. Our previous experimental results demonstrated that R‐CM@MSN@BC enhances tumor immunogenicity by inducing necroptosis of tumor cells and reshapes the immunosuppressive TME by reprogramming glutamine metabolism. Thus, we evaluated the combined efficacy of R‐CM@MSN@BC and anti‐PD‐L1 immunotherapy (**Figure** **7**A). Due to the immunosuppressive TME in CCA, administering anti‐PD‐L1 solely through the tail vein did not significantly inhibit primary and distant tumor growth. However, the group treated with R‐CM@MSN@BC + L displayed efficacy in suppressing primary tumor growth. Interestingly, the combination therapy of R‐CM@MSN@BC + L and anti‐PD‐L1 exhibited a substantial inhibition of bilateral tumor growth. Furthermore, mice in the R‐CM@MSN@BC + L plus anti‐PD‐L1 combination treatment group showed a more favorable prognosis with regard to survival (Figure 7B–F, Figure S8A–D, Supporting Information). Ki67 and H&E staining on primary and abscopal tumor tissues revealed a significant decrease in proliferating tumor cells in the R‐CM@MSN@BC + L plus anti‐PD‐L1 groups (Figure S8F, Supporting Information). These findings suggest that the combination of R‐CM@MSN@BC + L and anti‐PD‐L1 exhibits a synergistic therapeutic effect on tumor growth inhibition, as well as a promising abscopal effect. To gain further insights into the mechanisms underlying the enhanced antitumor activity, flow cytometry was employed to examine T cell infiltration in both primary (Figure 7G,H) and abscopal tumors (Figure 7I,J). In both primary and abscopal tumors, R‐CM@MSN@BC administration combined with anti‐PD‐L1 resulted in a significant increase in the proportions of cytotoxic T‐lymphocytes (CD3^+^CD8^+^ T cells). Subsequently, enzyme‐linked immunosorbent assays were conducted to assess alterations in the levels of several proinflammatory cytokines, including IL‐12, IL‐2, interferon‐γ, and tumor necrosis factor‐α, in mouse sera following the different treatments. As indicated by our results, R‐CM@MSN@BC can stimulate immune cells within the TME to secrete higher levels of inflammatory cytokines subsequent to irradiation (Figure S8G, Supporting Information). This study suggests that the combination therapy involving R‐CM@MSN@BC + L and anti‐PD‐L1 effectively stimulates a systemic antitumor immune response and enhances the efficacy of anti‐PD‐L1 in the treatment of CCA.

## 3 Conclusion

In conclusion, we have successfully developed a biomimetic controlled‐release drug nanosystem that integrates diagnostic imaging, photodynamic therapy, and immunotherapy. This nanosystem demonstrates the capacity to generate significant ROS and inhibit GSH production within tumor cells upon light exposure, leading to prolonged tumor cell eradication. Additionally, R‐CM@MSN@BC reshapes the immunosuppressive TME by reprogramming glutamine metabolism. The release of ROS from R‐CM@MSN@BC induces ICD effects, particularly through necroptosis in tumor cells. Simultaneously, the GLS1 inhibitor BPTES released promotes M1‐type macrophage polarization and inhibits T‐cell depletion. Through these pathways, R‐CM@MSN@BC effectively modifies the immunosuppressive TME, enhancing the adaptive immune response and creating a robust foundation for combined anti‐PD‐L1 immune checkpoint blockade therapy. The combination of R‐CM@MSN@BC with anti‐PD‐L1 therapy demonstrates significant efficacy in impeding the proliferation of both localized and metastatic tumors in murine models. This introduces a novel approach to hinder tumor recurrence and expansion. In essence, our research introduces a promising nanoparticle composite, referred to as the “trinity” nanoparticle, which holds potential for overcoming existing challenges in CCA management.

## 4 Experimental Section

### Materials

Tetraethyl orthosilicate (TEOS), bis[3‐(triethoxysilyl)propyl]tetrasulfide (BTESe), triethanolamine (TEA), fluorescein isothiocyanate (FITC), selenium (Se) powder, sodium (Na) power, sodium borohydride (NaBH_4_) and ammonium nitrate (NH_4_NO_3_) were purchased from Xi'an Ruixi Biotechnology Company, China. The list of primers, sequences of the siRNAs, antibodies for western blot, enzyme‐linked immunosorbent assay (ELISA) kits, and antibodies for CyTOF was provided in Tables S1 to S5 (Supporting Information), respectively.

### Clinical Samples

With the approval of CCA patients undergoing partial hepatectomy in the Center for Liver Transplantation and the Department of Hepatobiliary Surgery of Union Hospital, the paired tumor and surrounding non‐tumor tissues used in this study were obtained. All patients gave their informed consent. The research ethics committee of Union Hospital accepted all experimental protocols, and the same organization also awarded research ethics approval for this endeavor. The permission number of ethical approval statements was UHCT‐IEC‐SOP‐016‐03‐01.

### Cell Lines and Animals

The human bile duct epithelial cells (HIBEpic), human CCA cell lines (RBE, RBE, HuCCT1) and human normal liver cell lines (MIHA) were purchased from American Type Culture Collection (ATCC), and growed at 37 °C with 5% CO_2_ in a humidified atmosphere. The cells were grown in RPMI‐1640 (Gibco) supplemented with 10% FBS and 1% penicillin‐streptomycin at 37 °C in a humidified environment containing 5% CO_2_. Male C57BL/6 and Balb/c nude 4–6‐week‐old mice were also provided by the Vital River Laboratory Animal Technology Co. Ltd. and Beijing HFK Bioscience Co. Ltd., both of Beijing, China (Beijing, China). All animal studies and feeding procedures were carried out in compliance with the regulations established by the Animal Care Committee at Tongji Medical College, HUST, Wuhan, China. The permission number of ethical approval statements was [2020] IACUC Number: 2910. This research was carried out in accordance with the Helsinki declaration.

### Synthesis of BTESe

Bis [3‐(triethoxysilyl) propyl] diselenide (BTESe) was synthesized using a previously established method.^[^ ^31^ ^]^ In this procedure, a round‐bottomed flask was filled with a solution containing 2.37 g (30 mmol) of selenium powder, which was then placed in an ice‐water bath under a nitrogen atmosphere for a duration of 10 min. Subsequently, a carefully measured solution of NaBH_4_ (2.27 g, 60 mmol) dissolved in 25 ml of water was added dropwise to the selenium suspension. The resulting reaction mixture was stirred and allowed to react at 0 °C until the solution became colorless and completely dissolved the selenium powder. Subsequently, an additional quantity of selenium powder (2.37 g, 30 mmol) was introduced into the aforementioned solution, and the flask was subjected to heating at 100 °C for a duration of 10 min, resulting in the solution acquiring a reddish‐brown hue. Promptly thereafter, 12 grams of γ‐chloropropyl trimethoxysilane were swiftly added to the sodium diselenide stock solution, followed by overnight stirring. The resulting mixture was subjected to extraction using ethyl acetate, and subsequent rotary evaporation yielded an oily product. The crude product was subsequently purified through silica gel column chromatography, resulting in the formation of a dark yellow liquid product (3.32 g, ≈20% yield).

### Synthesis of Diselenide‐Bond Bridged Mesoporous Organosilica Nanoparticles

A mixture of 0.6 g of ethyltrimethylammonium tosylate (CTAT), 0.15 g of triethanolamine (TEA), and 40 mL of deionized water was stirred at 80 °C for 30 min. Subsequently, a dropwise addition of a solution containing 4.0 g of triethoxysilane (TEOS) and 1.0 g of BTESe was made to the surfactant solution. The resultant mixture was stirred at 80 °C and 1000 rpm for an additional 4 h. The product was collected through centrifugation, subjected to three washes with ethanol, and subsequently reflux‐treated in an ethanol solution of ammonium aminonitrate (1% w/v) for 12 h. Ultimately, the produced diselenide‐bridged mesoporous organosilica nanoparticles (MSN) was collected, washed, and dried for subsequent experimental use.

### Synthesis of MSN@BC

Diselenide‐bond bridged mesoporous organosilica nanoparticles solution with the concentration of 0.6 mg mL^−1^, and sonicated for well dispersion, subsequently, 50 mL of 1 mg mL^−1^ potassium permanganate (KMnO_4_, Sinopharm Chemical Reagent Co.) solution added slowly and continued to stir at room temperature for 30 min. Brown MSN were obtained after removing extra KMnO_4_ and free MnO_2_. For the preparation of MSN@BC, 100 µL 10 mg mL^−1^ Chlorin e6 (Ce6, Frontier Scientific, Inc.) dimethyl sulphoxide (DMSO, Sinopharm Chemical Reagent Co.) solution and 50 µL 2 mg mL^−1^ BPTES DMSO solution was added into 5 mg MSN. Stirred at room temperature for 14–16 h overnight, the precipitate was collected by centrifugation at 13 000 rpm and washed, re‐dispersed in deionized water to obtain MSN@BC. The above method was used to make Ce6 loaded MSN (MSN@C) and BPTES loaded MSN (MSN@B).

### Preparation of Erythrocyte Membranes Modified with RGD Peptides

In brief, 1 mL of fresh blood was withdrawn from the ophthalmic vein of male C57BL6/J wild‐type mice (20–22 g) through eyeball removal. This blood was then combined with 100 µL of EDTA anticoagulation solution. Erythrocytes were gathered via centrifugation at 720 g for 10 min. The obtained erythrocyte membranes were re‐suspended in 0.2 mm EDTA deionized water at a four‐fold volume to trigger membrane rupture. Following 60 min of hypotonic lysis, the samples were centrifuged at 20,000 g for 20 min to obtain erythrocyte membranes. The freshly acquired erythrocyte membranes were stored at 4 °C and utilized within a 6 h timeframe. Subsequently, DSPE‐PEG2000‐RGD (FITC) was introduced, and the sample were mixed by ultrasonic shaking. Capitalizing on the lipid affinity of DSPE and erythrocyte membranes, DSPE can spontaneously incorporate into the erythrocyte membranes' phospholipid bilayer. This process efficiently and non‐destructively facilitates the modification of RGD on the membrane surface.

### Preparation and Characterization of R‐CM@MSN@BC

To fabricate biomimetic MSN@BC nanosystem, erythrocyte membrane modified with RGD peptide was mixed with the same weight of MSN@BC in water and sonicated for 10 min in an ice bath. The above method was used to make R‐CM@MSN@B and R‐CM@MSN@C. The morphology, structure, and surface chemistry of these nanosystem were characterized by transmission electron microscope (TEM, TF20, FEI company, USA), UV–vis spectrophotometer (TU‐1810, BJPERSEE, China), X‐Ray photoelectron spectroscopy (XPS, K‐Alpha, Thermo Scientific, USA), N_2_ adsorption investigation (TriStar II 3020, Alpha technologies company, USA), and dynamic light scattering (DLS, NanoBrook 90plus PALS, Brookhaven, USA). Fluorescence spectrophotometer of the R‐CM@MSN@B, R‐CM@MSN@C, and R‐CM@MSN@BC were measured using a Fluoromax‐4 spectrofluorometer (Horiba Jobin Yvon Inc.) under 400 nm excitation. The concentration of Ce6 and BPTES in the supernatant was analysed by high performance liquid chromatography (HPLC, Agilent Technologies Inc., 1100S, USA) with a UV–vis detector at wavelength of 254 nm to calculate the encapsulation efficiency (EE) and drug loading capacity (DL) of Ce6 and BPTES.

To analyze erythrocyte membranes within nanosystems, Both erythrocyte membranes and various nanosystems were disrupted using RIPA buffer supplemented with a combination of PMSF and phosphatase inhibitor. The entire protein content was gathered through centrifugation and quantified utilizing the BCA protein assay kit. Proteins derived from both RBC membranes and diverse nanosystems were separated using SDS‐polyacrylamide gels, subsequently stained, and then captured through photography with the application of Coomassie blue solution. Detection of FITC‐RGD peptides on the surface of nanosystems by a spinning disk confocal super resolution microscope (SpinSR, OLYMPUS, Japan).

### Degradation Experiment and Drug Release Experiment In Vitro

R‐CM@MSN@BC was dispersed in PBS (pH 6.5, 7.4) solution with the concentration of 0.2 mg mL^−1^, respectively, among which pH 6.5 PBS solution, 200 µM H_2_O_2_ was added to simulate tumor microenvironment, pH 6.5 PBS solution with 200 µM H_2_O_2_ under laser irradiation was to imitate the therapeutic tumor microenvironment, and pH 7.4 PBS solution was to simulate in vivo. The resulting R‐CM@MSN@BC PBS solution was incubated separately at 37 °C in air bath shaker at 170 rpm. At the given time (0.5 h, 1 h, 3 h, 5 h, 7 h, 12 h, 1 d, 2 d, 3 d, and 5 d), 2 mL of above R‐CM@MSN@BC PBS solution was centrifuged. The cumulative degradation content of free silicon was determined using ICP‐OES in one portion of the supernatant, while the content of BPTES and Ce6 was determined using HPLC in the other.

### The Detection of Singlet Oxygen

To measure singlet oxygen, the decline absorption of 1,3‐diphenylisobenzofuran (DPBF) at 416 nm according to a reported literature. Firstly, nitrogen gas (N_2_) was blown into the PBS solution containing R‐CM@MSN@BC (50 µg mL^−1^) for 30 min to clear away the O_2_. Subsequently, 250 µM H_2_O_2_ was added to the R‐CM@MSN@BC solution above. After 30 min, 10 µg mL^−1^ DPBF DMSO solution was add into and exposed under 655 nm laser irradiation for certain time. After centrifugation, the absorbance of supernatant was recorded by the UV–vis spectrophotometer.

### Bioinformatic Analysis of RNA‐Seq Data for Human CHOL

From the TCGA database, the RNA‐seq and clinical information of various cancer classifications were obtained and these data were analyzed with the GEPIA tool.^[^ ^32^ ^]^ The survival (2.44.1) packages were used in R (4.1.0) to compute and construct survival curves.

### CRISPR/Cas9 Technique

Single guide RNAs (sgRNAs) of Control (sgControl), GLS1 (sgGLS1‐1^#^, sgGLS1‐2^#^ and sgGLS1‐3^#^) were designed and purchased from Merck (https://www.sigmaaldrich.cn/CN/zh). Then sgRNAs were cloned into lentiCRISPR v2 vector (#52 961, Addgene, USA). The sequences of sgRNAs were as flow, sgControl: CGCTTCCGCGGCCCGTTCAA; sgGLS1‐1^#^: AAACGCGTCCGTCTCCCCG; sgGLS1‐2^#^GACGCGTTTGGCAACAGCG; sgGLS1‐3^#^AGCACGCATCCGCAGCCCG.

### Cell Viability Assay

RBE cells were seeded into a 96‐well plate at 8000 cells per well and cultured for 24 h. Then, the cells were treated with PBS or R‐CM@MSN@BC. The cells were washed three times with PBS to eliminate non‐internalized chemicals after a 12 h incubation in the dark, and 200 µL of fresh culture media was added. Cells were irradiated with different intensities of 640 nm laser for the same time, or with the same intensity of 640 nm laser for different times, respectively. Cell viability was assessed using the CCK‐8 test (Beyotime, Shanghai, China) after an additional 24 h of incubation.

### Immunohistochemistry

GLS1, and Ki‐67 expression levels in tumor tissue were examined using immunohistochemical staining, as previously described,^[^ ^33^ ^]^ and images were taken using optical microscopy.

### H&E Staining

Images were taken using optical microscope after H&E staining to evaluate the histological alterations of tumors following various therapies, as previously described.^[^ ^34^ ^]^

### RNA Isolation and Quantitative Real‐Time PCR

Trizol was employed for the extraction of total RNA from the cells, and the NanoDrop ND‐1000 spectrophotometer, manufactured by NanoDrop Technologies, Thermo, was utilized for its quantification. PrimeScript RT Master Mix was employed to conduct reverse transcription on 0.5 µg of total RNA, enabling the synthesis of the first strand cDNA. For quantitative real‐time PCR, a Takara SYBR Green PCR Kit was employed. Each sample was subjected to analysis in triplicate wells. The siRNA sequence and primer details can be found in Table S1 and S2 (Supporting Information), respectively.

### Western Blot Analysis

The total protein content was obtained by employing a RIPA lysis buffer. The cytosolic protein and mitochondrial protein were extracted using a mitochondrial protein extraction kit and a nuclear protein extraction kit, respectively, following the guidelines provided by the manufacturer. The concentration of each sample was determined using a BCA protein assay kit. Equal concentrations of protein were loaded onto SDS‐PAGE and transferred onto PVDF membranes (Millipore, Bedford, USA), which were then blocked with 5% non‐fat milk for a duration of 1 h. Primary antibodies were incubated overnight at 4 °C. Following incubation with secondary antibodies conjugated to HRP, the immunoreactivity was detected utilizing an ECL substrate (BOSTER, Wuhan, China) and captured using an Image Lab imaging system. The comprehensive list of antibodies utilized can be found in table S3.

### Flow cytometry Analysis

According to the experimental method of previous,^[^ ^35^ ^]^ RBE cells after different treatments or tumor tissues were made into single‐cell suspensions. After co‐incubation with different fluorescent antibodies according to the manufacturer's instructions, the obtained cells were examined by flow cytometry (BD LSRFortessaX‐20 Special order product) and data were analyzed by a FlowJo software (Version 10). The list of antibodies were provided in Table S3 (Supporting Information).

### RNA‐Seq and Microarray Expression Data Analysis

The RNA from RBE cells subjected to various treatments was sequenced using next‐generation sequencing technology by Haplox Pharmaceutical Technology (Shenzhen, China). The software tools Hisat2 (version 2.2.1), htseq‐count, and edgeR were employed for mapping reads, calculating read counts, and analyzing differential expression, respectively.^[^ ^36^, ^37^, ^38^ ^]^ Subsequently, the data was normalized and analyzed using the limma package.^[^ ^39^ ^]^ Genes with a false discovery rate (FDR) less than 0.05 and an absolute fold change (|FC|) greater than 1.5 were identified as differentially expressed genes (DEGs).

### Time‐Of‐Flight Mass Cytometry (CyTOF) Analysis

Immune cells were obtained from CCA tumors utilizing the methodology outlined in Section Tumor Immune Microenvironment Assay using Flow Cytometry. These immune cells were subsequently labeled with antibodies containing metal tags. The antibody panel employed in this study comprised 42 antibodies conjugated with distinct metals (Table S5, Supporting Information). The evaluation of conjugated target molecule expression was conducted by detecting the metal signals utilizing the CyTOF system (Helios, Fluidigm, San Francisco, CA, USA).

### Transmission electron microscopy analysis

Transmission electron microscopy analysis were performed as previously described to observed cell morphology after different treatments.^[^ ^40^, ^41^ ^]^

### Nanosystems Assessment of 3D Tumor Cell Sphere Permeability

RBE cholangiocarcinoma cells were seeded in ultra‐low adsorption 96‐well plates at a density of 2 × 10^4^ cells per well and incubated for 24 h. Once the cells reached a spherical morphology, equal concentrations of MSN@BC, CM@MSN@BC, and R‐CM@MSN@BC nanosystems were added individually and incubated for an additional 24 h. The cell nuclei were then stained using Hoechst 33342, and the resulting cell spheres were observed and photographed at various levels using a confocal high‐intensity imaging system (ImageXpress Micro Confocal, Molecular Devices, USA).

### Internalization Efficacy Test

The cells were randomly divided into four groups and co‐incubated with R‐CM@MSN@BC (10 µg mL^−1^) for different time intervals (0 h, 2 h, 6 h, and 12 h) before being fixed with 4% paraformaldehyde for 35 min. The nuclei were located using Hoechst 33342 (Servicebio, Wuhan, China). Following three washes with PBS for five min each, confocal imaging was performed on the cell culture slides transferred to object slides. Subsequently, the ImageJ program was utilized to conduct relative semi‐quantitative fluorescence analysis in order to quantify the cellular uptake of micelles.

### ROS Generation in Vitro

Following the manufacturer's instructions, the fluorescent probe DCFH‐DA (Beyotime, Shanghai, China) was used to find ROS generation inside the cells following light exposure. On a 12‐well plate, 1 × 10^5^ RBE cells were planted. The culture medium was taken out and thoroughly washed with PBS twice after the cells had been attached. R‐CM@MSN@BC were co‐incubated with RBE cells for 6 h before DCFH‐DA was loaded into the cells. Cells were given two PBS washes after 20 min of incubation before being exposed to light at a power density of 300 mW cm^−2^. After irradiation, fluorescence microscope and flow cytometer were used to measure the intensity of the fluorescence.

### Hypoxia Probe Staining

The Hypoxyprobe‐1 plus kit (Hypoxyprobe, Burlington, MA) was used to identify the hypoxic region in the xenografted tumor following the manufacturer's instructions. Briefly, nude mice bearing tumors were injected with 60 mg k^−1^g of pimonidazole hydrochloride intravenously, following 640 nm irradiation (300 mW cm^−2^, 3 min). The tumors were removed, cut into sections, and subjected to immunohistochemistry analysis after 90 min. In accordance with the protocol, mouse monoclonal antibody was used to examine frozen tissue slices. DAPI was used to label the cell nuclei.

### Live/Dead cell staining

In accordance with manufacturer's instructions, Calcein/PI Cell Viability/Cytotoxicity Assay Kit (Beyotime, Shanghai, China) were utilized to assess further R‐CM@MSN@BC‐mediated chemo‐PDT effects. RBE cells were co‐incubated with the nanoparticles for 4 h before being washed three times with PBS for 3 min, exposed to laser irradiation (640 nm, 300 mW cm^−2^, 3 min), and then incubated with Calcein‐AM (2 µM) and PI (5 µM) at 37 °C for 20 min before being photographed under a fluorescence microscope.

### ELISA Analysis

Following the manufacturer's instructions, the appropriate ELISA Assay Kits (Beyotime, Shanghai, China) were used to measure the levels of IFN‐γ, IL‐12, IL‐2 and TNFα in the serum of treated C57BL/6 mouse after various treatments. The list of elisa kit was provided in Table S4 (Supporting Information).

### Immunofluorescence Staining

Following the previous protocol,^[^ ^34^ ^]^ immunofluorescence assays were used to evaluate the level of membrane translocation of CRT and the extent of HMGB1 leakage in RBE cells after different treatments. Also was used to assess the degree of infiltration of CD4^+^ T cells, CD8^+^ T cells, in tumor tissues of mice after different treatments.

### In Vivo Imaging

The fluorescence imaging and MRI were studied in RBE tumor‐bearing Balb/c nude mice. The mice were intravenously injected with the R‐CM@MSN@BC and then, imaged by a fluorescence imaging instrument (In‐Vivo FX PRO, Bruker, Germany) or an MRI scanner (4.7 T Bruker BioSpec, Bruker, Germany) belonged to National Center for Magnetic Resonance in Wuhan, Innovation Academy for Precision Measurement Science and Technology, CAS. The signal intensity was achieved by built‐in software of the imaging systems.

### Metabolites Assay In Vitro

Following the manufacturer's instructions, the intracellular metabolites level in RBE cells cultured in different treatment medium for 24 h was measured using a GSH and GSSG Assay Kit (Beyotime, Shanghai, China), NADP^+^/NADPH Assay Kit with WST‐8 (Beyotime, Shanghai, China), Micro Glutamic Acid (Glu) Content Assay Kit (Solarbio, Beijing, China), LA Assay Kit (Solarbio, Beijing, China).

### In Vivo Experiments

In order to assess the inhibitory effect of photodynamic therapy (PDT) mediated by R‐CM@MSN@BC on tumors in mice, a subcutaneous injection of 5 × 10^6^ RBE cells resuspended in saline was administered to the right flanks of BALB/c nude mice. The mice were then randomly assigned to one of four groups: saline, R‐CM@MSN@BC (10 µg mL^−1^), R‐CM@MSN@C (10 µg mL^−1^) with laser irradiation (+), R‐CM@MSN@B (10 µg mL^−1^) with laser irradiation (+), or R‐CM@MSN@BC (10 µg mL^−1^) with laser irradiation. This randomization occurred once the average tumor volume reached ≈100 mm^3^. Intravenous administration of all medications was performed, and subsequent measurements of body weight and tumor volume were recorded. The tumor volume was calculated by tumor length × tumor width × tumor width /2. Furthermore, the survival time of the BALB/c nude mice with tumors (n = 5 per group) was recorded daily for a period of 30 days. Histological changes, proliferation, and apoptosis levels of the tumors were assessed using H&E staining and the TUNEL assay, respectively, to confirm the efficacy of the R‐CM@MSN@BC treatment. Representative tumor tissues were also photographed.

To verify the effect on tumor microenvironment after R‐CM@MSN@BC treatment, a C57BL/6 mice model was constructed with subcutaneous graft tumor. Briefly, an AKT/Notch intracellular domain (NICD) was first obtained in situ intrahepatic cholangiocarcinoma model (purchased from Shouzheng Pharma Biotechnology Co., Ltd.),^[^ ^42^ ^]^ then mice were executed and well‐grown tumor tissues were selected and placed in sterile glass dishes. tumor tissues in sterile glass dishes, and the tumor tissues were cut up to ≈1 mm^3^. Next, the skin of the right leg of C57BL/6 mice was shaved off, disinfected and the skin of the right leg of the mice was cut, and the tumor tissue block was implanted under the skin of the right leg of C57BL/6 mice. Finally, the skin incision of the mice was sutured and again. After the subcutaneous tumor volume grew to about 100 mm^3^, the mice were randomly divided into three groups (5 mice per group) and treated with saline, R‐CM@MSN@C (10 µg mL^−1^) with laser irradiation (+), and R‐CM@MSN@BC (210 µg mL^−1^) with laser irradiation (+), respectively. On day 14, the mice were executed and collected their tumor tissues and evaluated the immune cell infiltration in the tumor tissues by CyTOF.

In order to assess the inhibitory effect of R‐CM@MSN@BC in combination with anti‐PD‐L1 treatment on distant septal tumors in mice, bilateral subcutaneous C57BL/6 tumor models were established. Once the tumor volume reached an average size of ≈100 mm^3^, the tumor‐bearing mice were randomly divided into four groups (n = 5 per group): (1) Saline; (2) anti‐PD‐L1; (3) R‐CM@MSN@BC + light; and (4) R‐CM@MSN@BC + light + anti‐PD‐L1. The changes in volume of the bilateral tumors were measured, and the tumors were dissected and photographed on day 25 after receiving the respective treatments. Ki‐67 and HE staining of tumor tissues were also performed to evaluate the inhibition of primary and distant tumors in mice by different treatment modalities.

### Statistical Analysis

All measurements were provided as mean standard deviation (SD). The quantitative data was evaluated using the Student's t‐test or the Mann‐Whitney U test. To compare the two groups statistically in paired samples, the Wilcoxon signed‐rank test or the paired t‐test were used. The count data was examined using the Pearson Chi‐Square test. The appropriate test was used for ranking data, either the Kruskal‐Wallis test or the Mann‐Whitney U test. The repeated measures ANOVA was used to assess the differences in tumor volume between groups. To analyze the statistical variances between survival curves, the log‐rank test was used. Prism 8.0 (GraphPad, San Diego, USA) was used to estimate the survival curves. The R software version 4.1.0 (Auckland, NZ) for all testing (*P < 0.05, **P < 0.01, ***P < 0.001) was used.

## Conflict of Interest

The authors declare no conflict of interest.

## Supporting information

Supporting Information

## Data Availability

The data that support the findings of this study are available from the corresponding author upon reasonable request.
