# Supplementary material for: Necroptosis‐Mediated Synergistic Photodynamic and Glutamine‐Metabolic Therapy Enabled by a Biomimetic Targeting Nanosystem for Cholangiocarcinoma
Source: Adv Sci (Weinh). 2024 Jun 5;11(29):2309203. doi: 10.1002/advs.202309203 (PMC11304281; doi:10.1002/advs.202309203)
Supplement: Supplementary file 1 — Supporting Information [file ADVS-11-2309203-s001.pdf]

ADVANCED SCIENCE
Open Access

Supporting Information

for *Adv. Sci.*, DOI 10.1002/advs.202309203

Necroptosis-Mediated Synergistic Photodynamic and Glutamine-Metabolic Therapy Enabled by a Biomimetic Targeting Nanosystem for Cholangiocarcinoma

Qichang Zheng, Tianhao Zou, Weimin Wang, Chen Zhang, Shaobo Hu, Xiang Cheng, Ran Liu, Guoliang Wang, Ping Sun, Xing Zhou, Bing Yang, Jianjun Xu, Yang Gao* and Jinyang Gu**

Supporting Information

Title

Necroptosis-Mediated Synergistic Photodynamic and Glutamine-Metabolic Therapy Enabled by a Biomimetic Targeting Nanosystem for Cholangiocarcinoma

Qichang Zheng [1, 2†]， Tianhao Zou [1†]， Weimin Wang [2†]， Chen Zhang [1]， Shaobo Hu [1]， Xiang Cheng [3]， Ran Liu [1], Guoliang Wang [1], Ping Sun [2]， Xing Zhou [2]， Bing Yang [2], Jianjun Xu [1]， Yang Gao [2*]， Jinyang Gu [1*]*

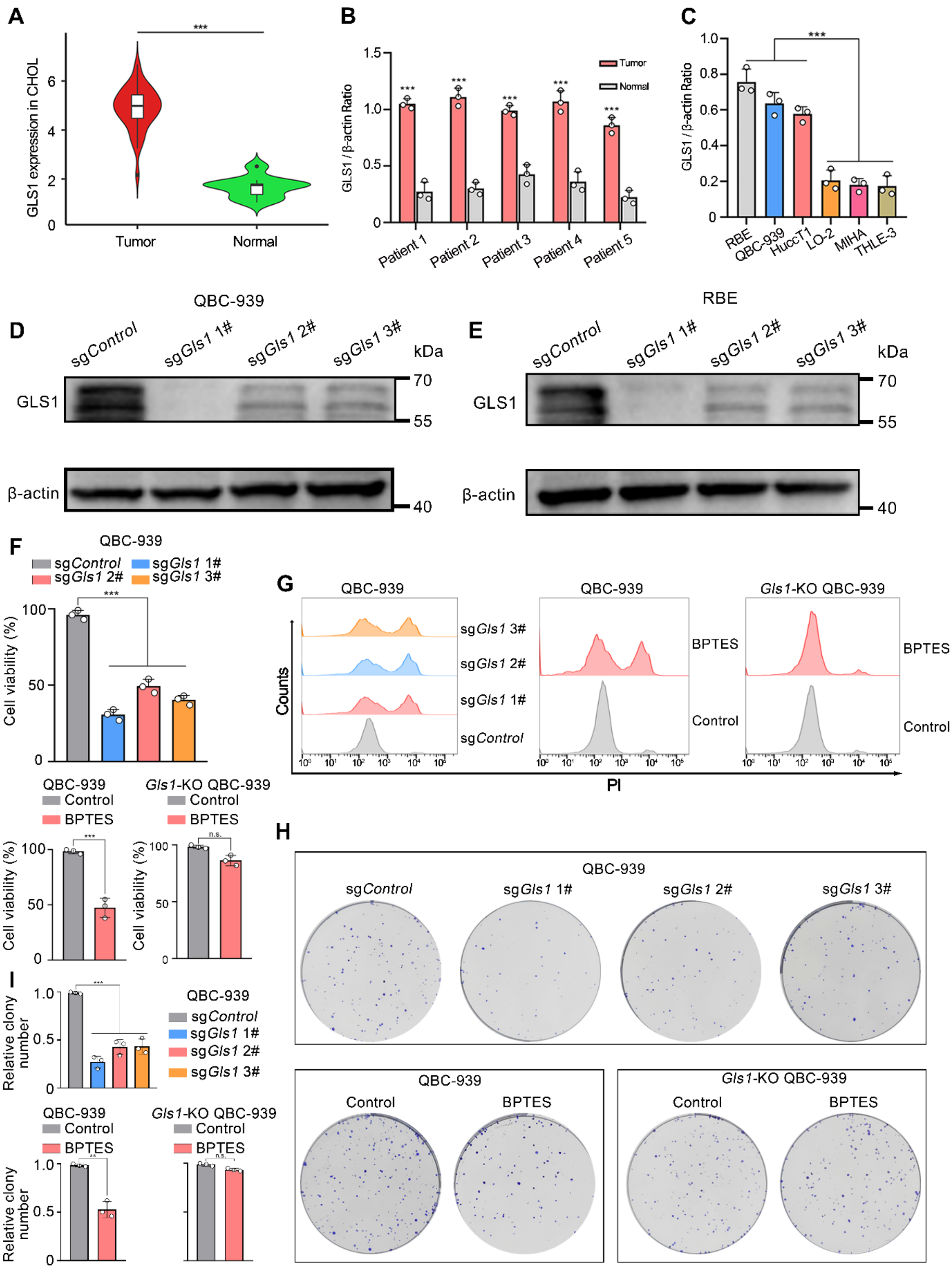

Figure S1. (**A**) Differential analysis of GLS1 mRNA expression in cholangiocarcinoma from TCGA and GTEx datasets. (**B**) Qualification of protein bands in Figure 1D. (**C**) Qualification of protein bands in Figure 1F. (D, E) PRMT5 was knocked out in QBC-

939 and RBE cells by CRISPR/Cas9. Western blot analysis was repeated three times independently. (**F**) The viability of RBE cells or GLS1-KO RBE cells after different treatment. (**G**) Flow cytometry analysis of dead cells in RBE cells or GLS1-KO RBE cells after different treatments. (**H, I)** Representative images and its corresponding quantitative analysis of the RBE cells or GLS1-KO RBE cells after different treatment, assessed with colony-forming assay. n=3 per group n.s., no significance; * P <0.05; ** P <0.01; *** P <0.001.

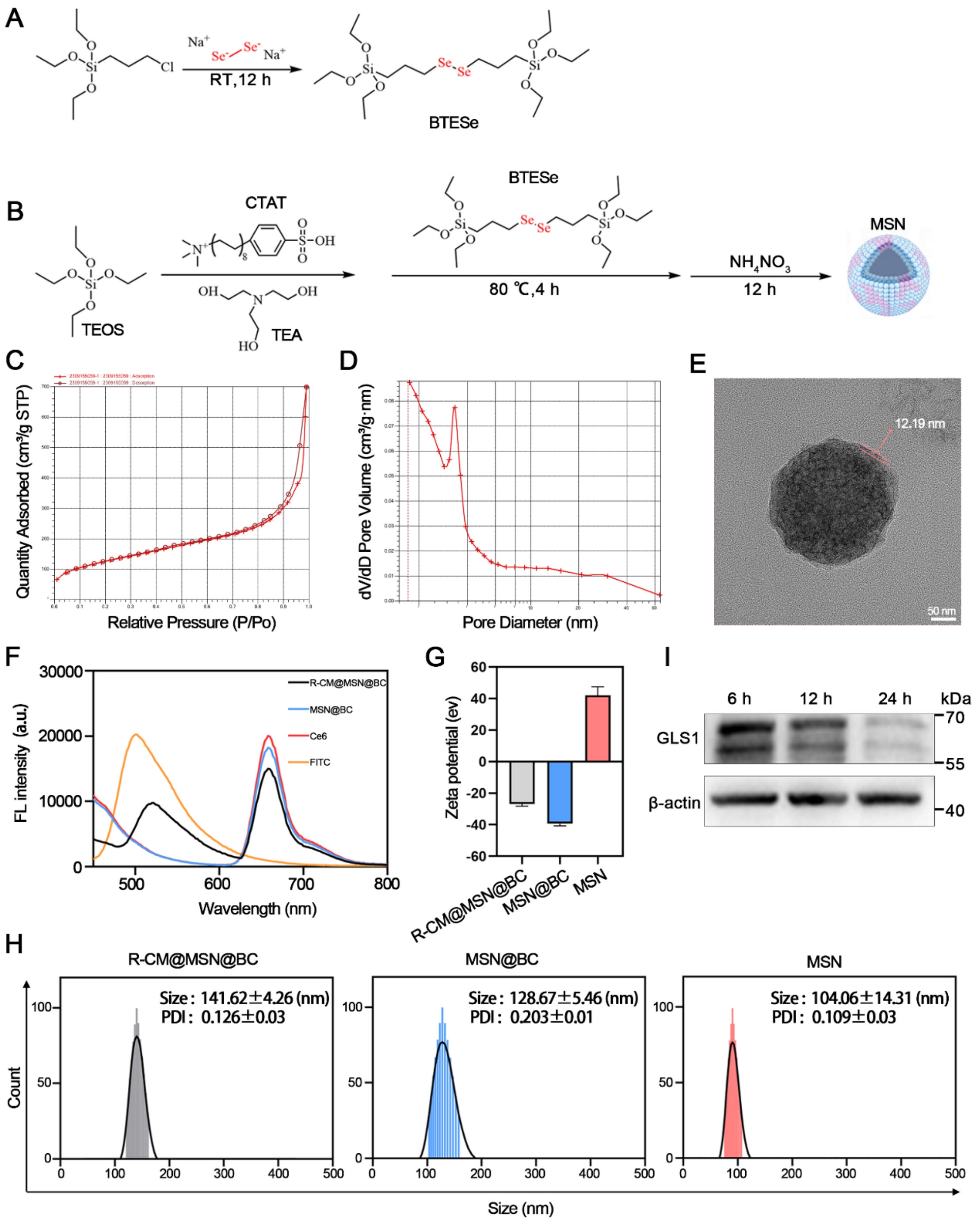

Figure S2. (A) The equation for the chemical synthesis of bis[3-(triethoxysilyl)propyl]diselenide (BTESe). **(B)** The equation for the chemical synthesis of diselenide bond containing hollow mesoporous organosilica nanoparticle (MSN). **(C)** Nitrogen adsorption-desorption isotherms of MSN. **(D)** Pore size distribution of MSN. **(E)** Representative TEM images of R-CM@MSN@BC, the thickness of erythrocyte coating is about 12.19 nm. Scale bar, 50 nm. **(F)** Fluorescence spectra of FITC fluorescent probe, photosensitizer Ce6, MSN@BC and R-CM@MSN@BC. **(G,**

H) The diameter and zeta potentials of MSN, MSN@BC, R-CM@MSN@BC measured by DLS; n=3 per group. **(I)** Western blot images of GLS1 proteins in mouse tumor tissues after injection of the nanosystems and 5-minute light stimulation.

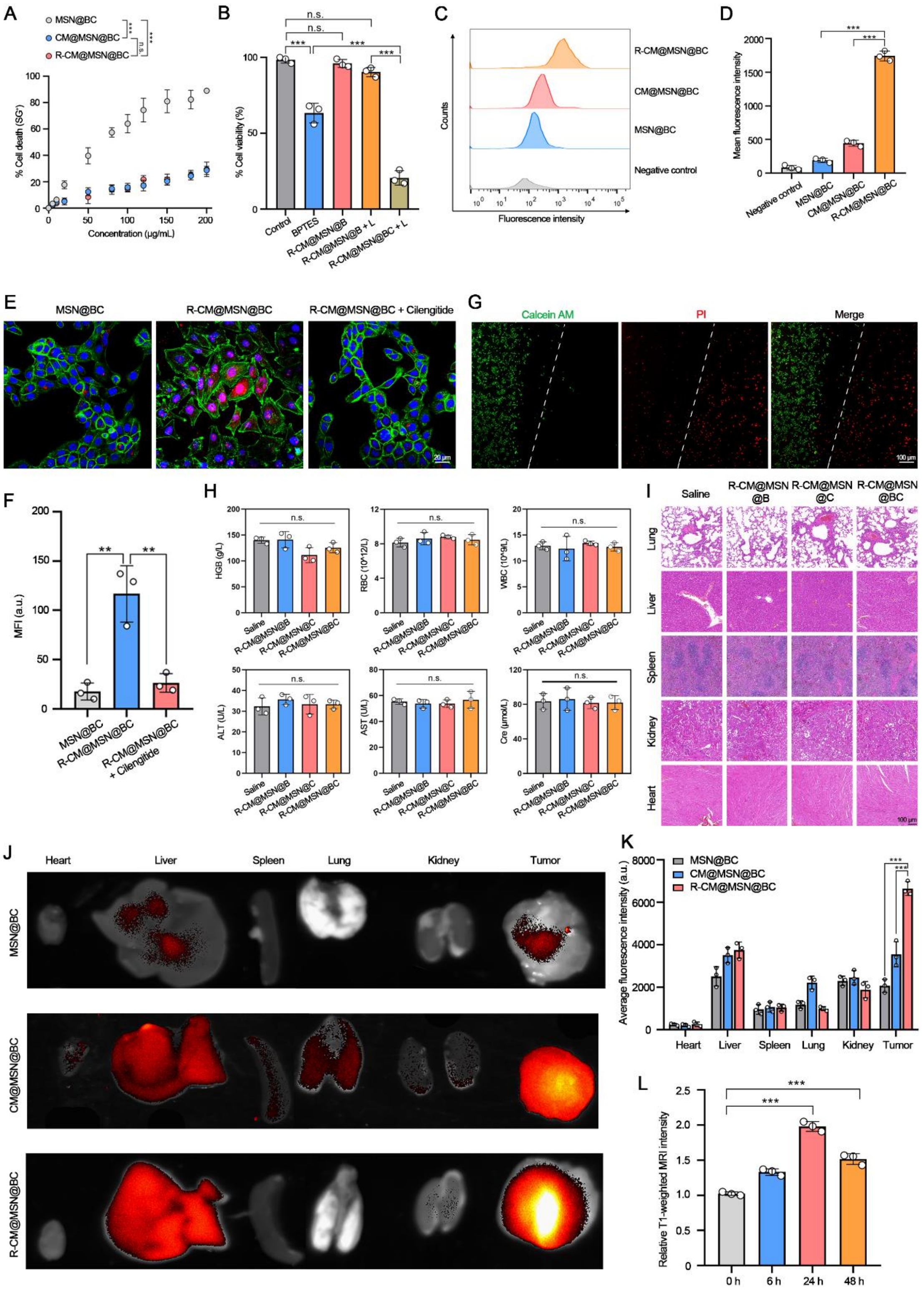

Figure S3. (A) RBE cells were treated with different nanosystem for indicated concentration, cell death was measured by SytoxGreen positivity assay; n=3 per group. **(B)** Cell viability of RBE cells after different treatment, cell viability was measured by CCK8 assay; n=3 per group. **(C, D)** Analysis of the fluorescence intensity of RBE cells using flow cytometry after they were incubated with the different nanosystem (10 μg/mL) for 6 h; n=3 per group. **(E, F)** CLSM images and relative semi-quantitative analysis of the fluorescence intensity of RBE cells after incubated with different nanosystem (10 μg/mL) for 6 h; n=3 per group. Red fluorescence is emitted by Ce6 in the nanosystem, blue fluorescence label the nuclei and green fluorescence label the cytoskeleton. Scale bar: 20 μm. **(G)** Fluorescence images of cells after incubation with R-CM@MSN@BC, then with or without irradiation (red and green indicate nonviable and viable cells, respectively). The areas to the right of white dotted lines were irradiated. Scale bars: 100 μm. **(H, I)** Biochemical indicator tests and histological examination (H&E) staining images of various organ slices from C57BL/6 mice after different treatments; n=3 per group. Scale bar: 100 μm. **(J, K)** Fluorescence imaging and its quantitative analysis of mouse tumors and their vital organs by small animal live imaging system, experiments were performed 48h after tail vein injection of different nanosystem; n=3 per group. **(L)** Quantification of average relative T1-weighed MRI intensity in the tumors after i.v. injection of R-CM@MSN@BC by time; n=3 per group. n.s., no significance; * $P < 0.05$; ** $P < 0.01$; *** $P < 0.001$.

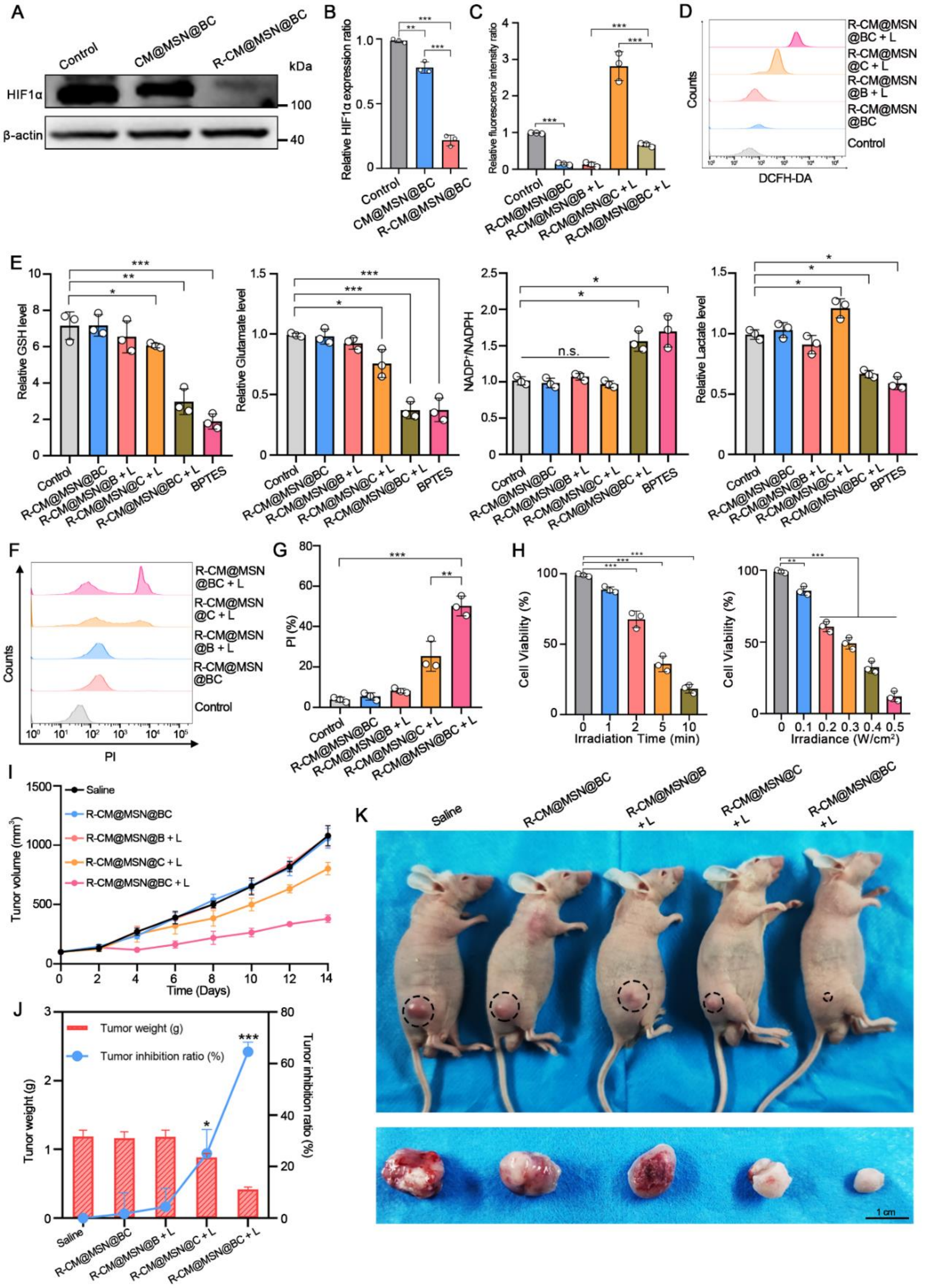

Figure S4. (A, B) Western blot images and quantification analysis of HIF-1α proteins after co-incubation with CM@MSN@BC (10 μg/mL) or R-CM@MSN@BC (10 μg/mL) for 6 h; n=3 per group. **(C)** Analysis of ROS production in RBE cells after different treatments by flow cytometry. **(D)** Quantitative analysis of metabolite content

(GSH, Glutamate, $NADP^+$/NADPH and Lactate) in RBE cells after different treatments; n=3 per group. **(E, F)** Qualitative and quantitative analysis of dead cells in RBE cells after different treatments by flow cytometry; n=3 per group. **(G)** RBE cells were co-incubated with R-CM@MSN@BC for 6 h, and the cell viability of RBE cells was measured after different light times or irradiance. **(H)** Average tumor growth kinetics of mice receiving the indicated treatments. **(I)** Tumor weight and inhibition ratio of various treated mice (n=5). **(J)** Representative images of tumor-bearing mice and harvested tumors after various treatments. Scale bar: 1 cm. n.s., no significance; * P <0.05; ** P <0.01; *** P <0.001.

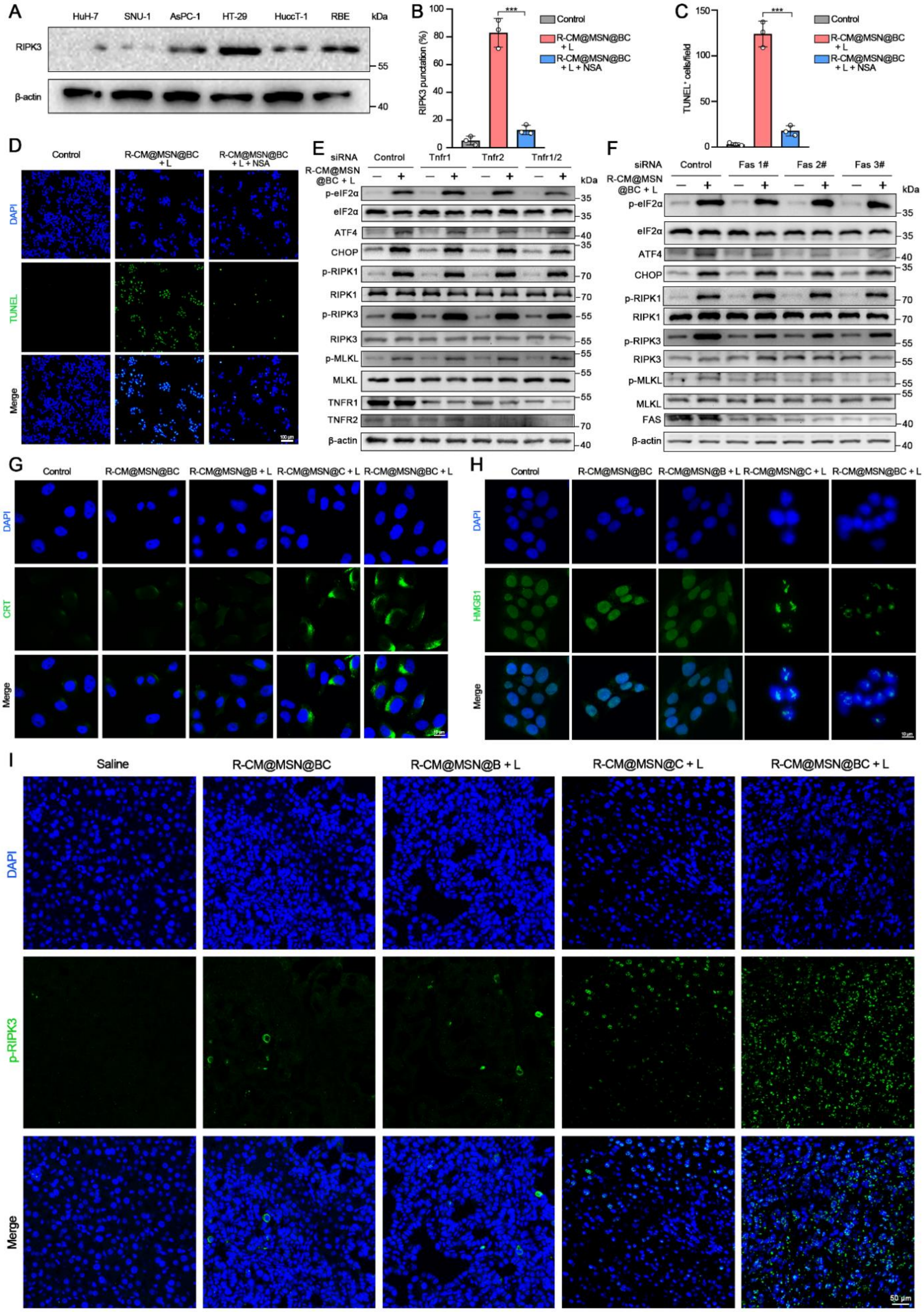

Figure S5. (A) Protein expression levels of RIPK3 in different cell lines, Huh-7 and SNU-1 as negative control, Aspc-1 and HT-29 as positive control. The levels of

indicated proteins were determined by immunoblotting. n=3 independent biological repeats. (**B**) Quantitative analysis of RIPK3 punctation level in Fig. 6F; n=3 per group. (**C, D**) TUNEL assays were performed on RBE cells from different treatments. DAPI for nuclei. Representative images are shown. Microscopic quantification of TUNEL-positive cells was shown on the right.; n=3 per group. (**E**) RBE cells were transfected (48 h) with control siRNA, Tnfr1 siRNA, Tnfr2 siRNA or Tnfr1/2 siRNA, then treated with PBS and R-CM@MSN@BC plus laser, respectively. The levels of indicated proteins were determined by immunoblotting. n=3 independent biological repeats. (**F**) RBE cells were transfected (48 h) with control siRNA, or sequentially distinct Fas siRNA, then treated with PBS and R-CM@MSN@BC plus laser, respectively. The levels of indicated proteins were determined by immunoblotting. n=3 independent biological repeats. (**G, H**) Fluorescent images of RBE cells that experienced different treatments for detecting calreticulin (CRT) and high mobility group box 1 (HMGB1) expression levels. Scale bar: 10 μm. (**I**) Immunostaining for p-RIPK3 on tumor sections was performed (n=5 mice in each group). DAPI for nuclei. Representative images are shown. Microscopic quantification of p-RIPK3–positive cells is shown at the bottom. n.s., no significance; * $P < 0.05$; ** $P < 0.01$; *** $P < 0.001$.

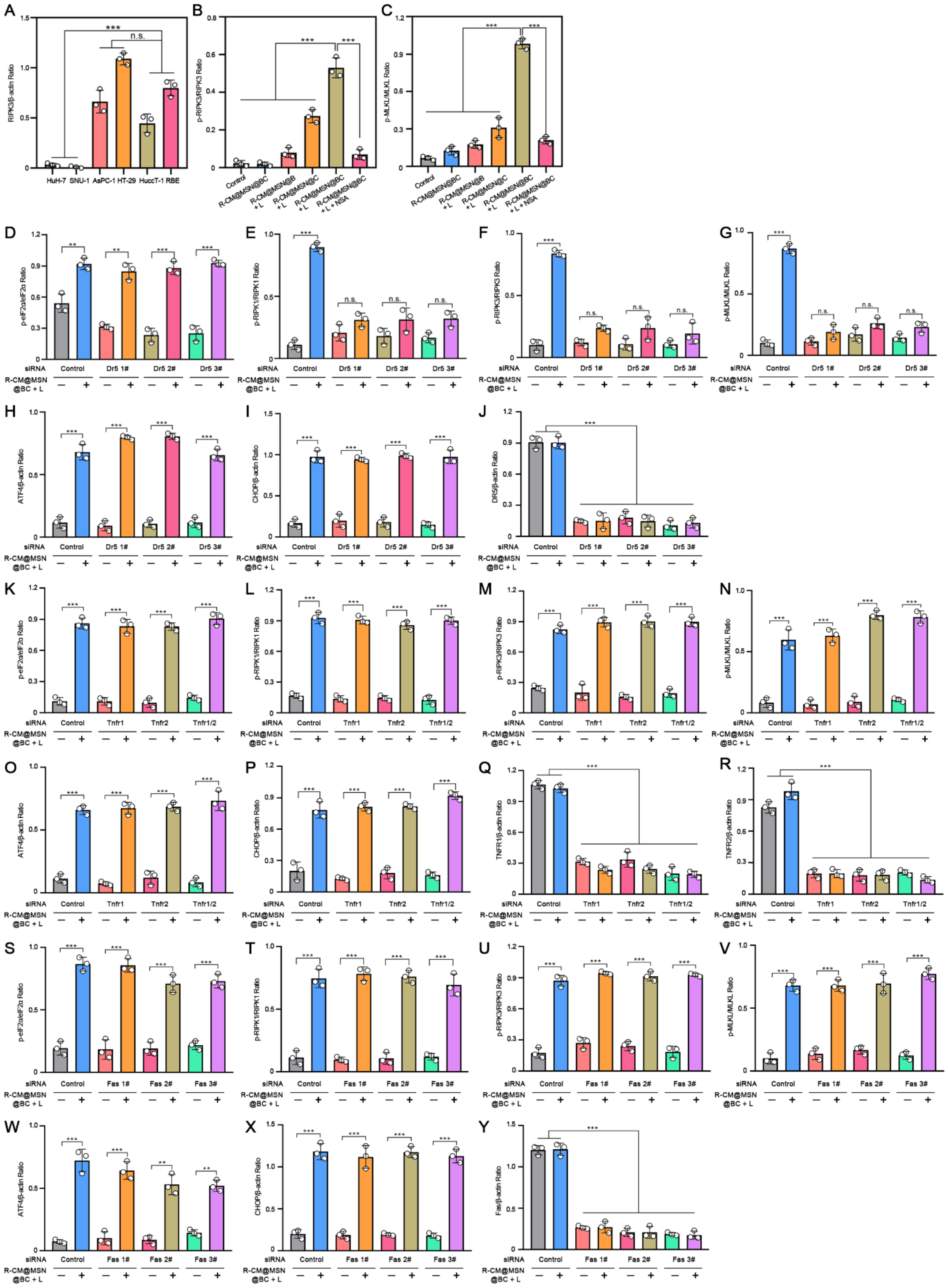

Figure S6. Qualification of protein bands in Figure 6 and Figure S4. Data were expressed as the mean ± SEM; n=3 per group. n.s., no significance; * P <0.05; ** P <0.01; *** P <0.001.

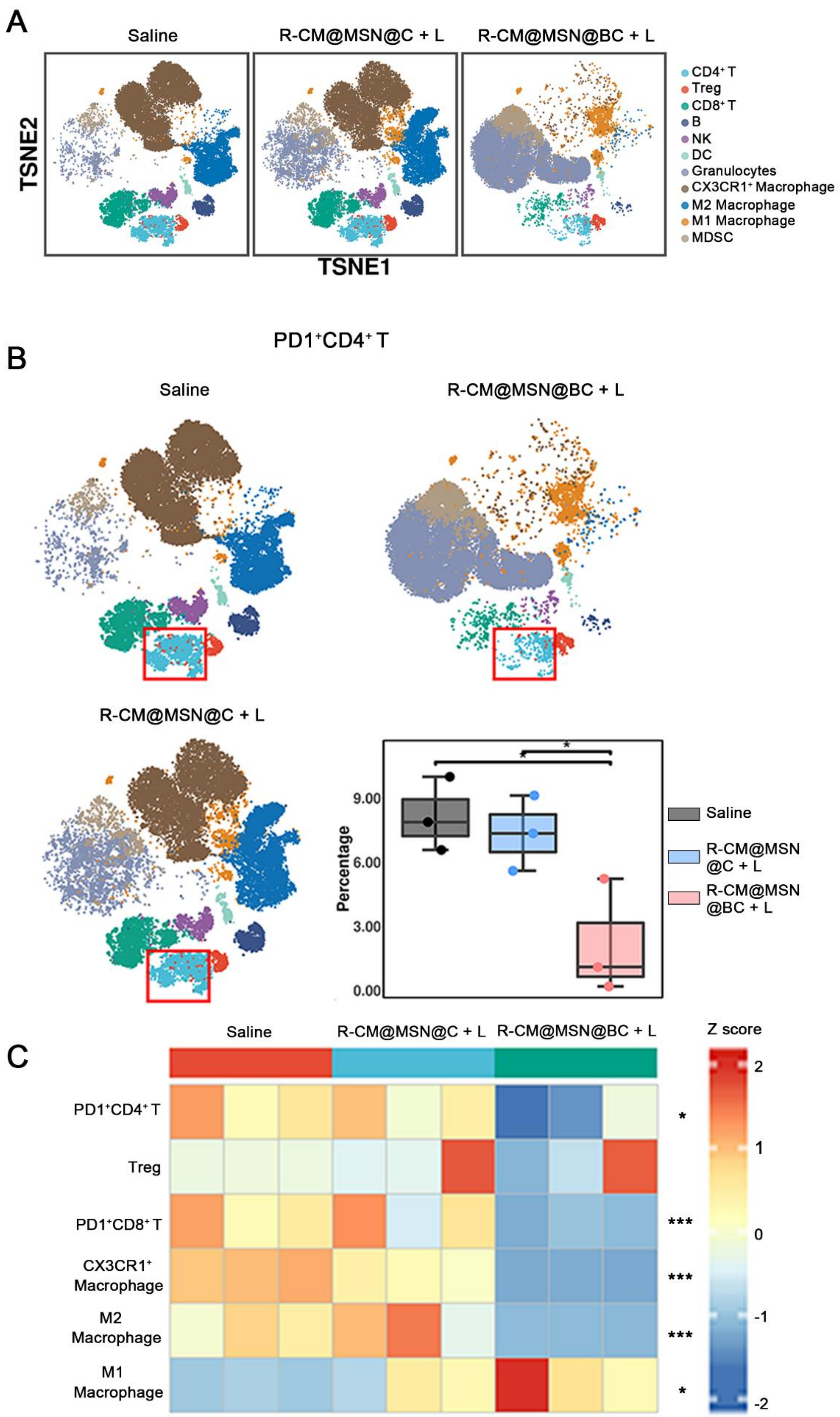
A
Saline
R-CM@MSN@C + L
R-CM@MSN@BC + L
TSNE2
TSNE1
CD4⁺ T
Treg
CD8⁺ T
B
NK
DC
Granulocytes
CX3CR1⁺ Macrophage
M2 Macrophage
M1 Macrophage
MDSC
B
PD1⁺CD4⁺ T
Saline
R-CM@MSN@BC + L
R-CM@MSN@C + L
Percentage
9.00
6.00
3.00
0.00
Saline
R-CM@MSN @C + L
R-CM@MSN @BC + L
C
Saline
R-CM@MSN@C + L
R-CM@MSN@BC + L
Z score
PD1⁺CD4⁺ T
Treg
PD1⁺CD8⁺ T
CX3CR1⁺ Macrophage
M2 Macrophage
M1 Macrophage
2
1
0
-1
-2

Figure S7. (**A**) A t-distributed stochastic neighbor embedding (t-SNE) plot via nonlinear dimensionality reduction showing the immune cell clusters in the different treatments tumors. (**B**) t-SNE plots showing expression levels of $PD1^{+}CD4^{+}$ T cells with corresponding quantitative comparisons. (**C**) A heatmap showing the differential expression of 6 cell clusters by CyTOF. n=3 per group. n.s., no significance; * P <0.05; ** P <0.01; *** P <0.001.

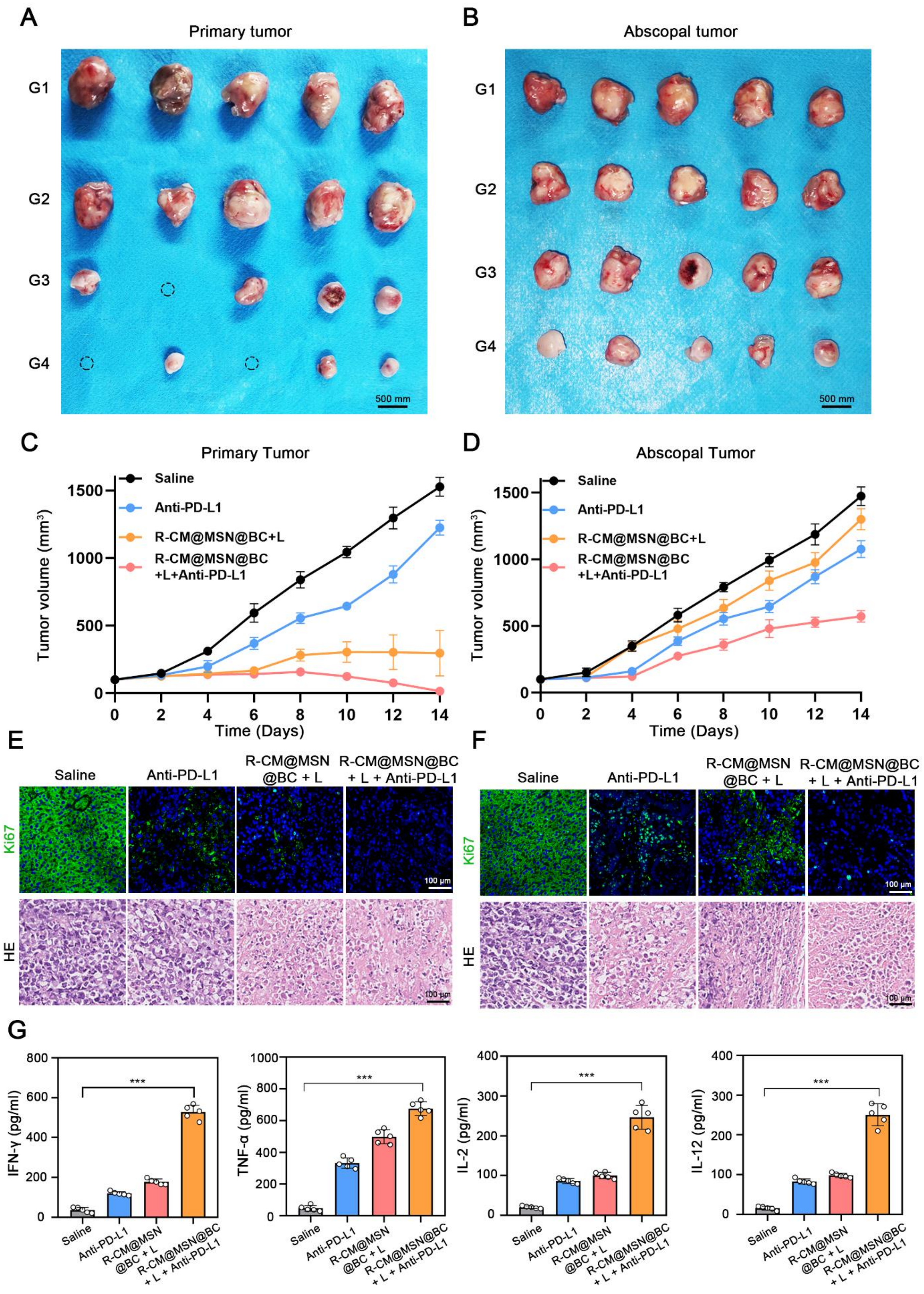

Figure S8. (**A, B**) Primary and distant tumor images in the different groups after the indicated treatments (n=5). Scale bar, 500 mm. G1, Saline; G2, Anti-PD-L1; G3, R-

CM@MSN@BC + L; and G4, R-CM@MSN@BC + L + Anti-PD-L1. (**C, D**) Average primary and abscopal tumor growth kinetics of mice receiving the indicated treatments. (**E, F**) H&E and Ki67 staining images of primary and abscopal tumor harvested from C57BL/6 mice subcutaneous graft tumor model after different treatments (Scale bar: 100 μm). (**G**) Elisa analysis of IFN-γ, IL-2, IL-12 and TNF-α of tumors after different treatments. n =5 per group. n.s., no significance; * $P < 0.05$; ** $P < 0.01$; *** $P < 0.001$.

Table S1. Primer list for qRT-PCR.

Primer	Applied Biosystems
GLS1	Forward: CACTCAAATCTACAGGATTGCG<br>Reverse: CCAGACTGCTTTTTAGCACTTT
β-actin	Forward: CATGTACGTTGCTATCCAGGC<br>Reverse: CTCCTTAATGTCACGCACGAT

Table S2. Sequences of the siRNAs used in this study.

Gene name	Sequence
Tnfr1	CATTGGTTTAATGTATCGCTA
Tnfr2	GCCTCACTTGCCTGCCGATAA
Dr5 1#	GCAGTCTCATTTGCACCCATA
Dr5 2#	CCACAAAGAATCAGGTACAAA
Dr5 3#	CTCACTGGAATGACCTCCTTT
Fas 1#	CTATCATCCTCAAGGACATTA
Fas 2#	GTTGCTAGATTATCGTCCAAA
Fas 3#	GTGCAGATGTAAACCAAACTT

Table S3. Antibody list for western blot.

Antibody	Company	Catalog No.	Application	Dilution
Anti-β-Actin (Mw of β-Actin = 45 kDa)	Cell Signaling Technology	3700	WB	1:1000

Anti-Glutaminase-1/GLS1 (Mw of GLS1 = 55-65 kDa)	Cell Signaling Technology	56750	WB, IHC	1:1000
Anti- HIF-1α (Mw of HIF-1α= 120 kDa)	Cell Signaling Technology	36169	WB	1:1000
Anti- RIP1 (Mw of RIP1= 78 kDa)	Cell Signaling Technology	73271	WB	1:1000
Phospho-RIP (Ser166) Anti-Phospho-RIP (Ser166) (Mw of Phospho-RIP)= 78-82 kDa)	Cell Signaling Technology	44590	WB	1:1000
Anti- RIP3 (Mw of RIP3 = 60 kDa)	Cell Signaling Technology	10188	WB	1:1000
Anti-Phospho-RIP3 (Ser227) (Mw of Phospho-RIP3 = 46-62 kDa)	Cell Signaling Technology	93654	WB	1:1000
Anti-MLKL (Mw of MLKL = 54 kDa)	Cell Signaling Technology	26539	WB	1:1000
Anti- Phospho-MLKL (Ser358) (Mw of Phospho-MLKL = 54 kDa)	Cell Signaling Technology	18640	WB	1:1000
Anti- eIF2α (Ser51) (Mw of eIF2α = 38 kDa)	Cell Signaling Technology	5324	WB	1:1000
Anti- Phospho-eIF2α (Ser51) (Mw of Phospho-eIF2α = 38 kDa)	Cell Signaling Technology	3398	WB	1:1000
Anti- ATF4 (Mw of ATF4 = 49 kDa)	Cell Signaling Technology	11815	WB	1:1000
Anti- CHOP (Ser51)	Cell Signaling Technology	2895	WB	1:1000

(Mw of CHOP =27 kDa)				
Anti- DR5 (Mw of DR5 = 48 kDa)	Cell Signaling Technology	8074	WB	1:1000
Anti- Fas (Mw of Fas = 40-50 kDa)	Cell Signaling Technology	8023	WB	1:1000
Anti- TNF-R1 (Mw of TNF-R1 = 55 kDa)	Cell Signaling Technology	3736	WB	1:1000
Anti- TNF-R2 (Mw of TNF-R2 = 60-80 kDa)	Cell Signaling Technology	72337	WB	1:1000
FITC anti-mouse CD3 Antibody	Biolegend	100203	Flow	1:500
PE anti-mouse CD8b Antibody	Biolegend	126607	Flow	1:500

Table S4. ELISA kit in the study.

Cytokine ELISA kit	Company	Catalog No.
Mouse IFN-γ ELISA Kit	Abcam	ab282874
Mouse TNF-α ELISA Kit	Abcam	ab208348
Mouse IL-2 ELISA Kit	Abcam	ab100706
Mouse IL-12 ELISA Kit	Abcam	ab119531

Table S5. List of Antibodies in CyTOF

List	Channel	Epitope	Brand	Category number
1	89Y	CD45	Biolegend	103102
2	115In	CD3ε	Biolegend	100302
3	139La	Ki-67	eB	14-5698-82
4	141Pr	CD24	Biolegend	101802
5	142Nd	CD172a(SIRPα)	Biolegend	144002
6	143Nd	Granzyme B Recombinant	Biolegend	372202
7	144Nd	CX3CR1	Biolegend	149002

8	145Nd	Gr-1	Biolegend	108402
9	146Nd	CD279(PD-1)	Biolegend	135202
10	147Sm	Ly-6G	Biolegend	127602
11	148Nd	Ly-6C	Biolegend	128002
12	149Sm	CD64	Biolegend	139302
13	150Nd	CD44	Biolegend	103002
14	151Eu	CD62L	Biolegend	104402
15	152Sm	CD11c	Biolegend	117302
16	153Eu	Siglec-F	BD	552125
17	154Sm	FoxP3	eB	14-5773-82
18	155Gd	CD38	Biolegend	102702
19	156Gd	CD194(CCR4)	Biolegend	131202
20	157Gd	CD366(Tim-3)	Biolegend	119702
21	158Gd	CD19	Biolegend	115502
22	159Tb	F4/80	Biorad	MCA497G
23	160Gd	TCRb	Biolegend	109202
24	161Dy	CD49b(pan-NK cells)	Biolegend	108902
25	162Dy	CD183(CXCR3)	Biolegend	126502
26	163Dy	CD25(IL-2Rα)	Biolegend	101902
27	164Dy	CD103	Biolegend	121402
28	165Ho	CD95(Fas)	Biolegend	152602
29	166Er	CD27	Biolegend	124202
30	167Er	CD206	Biolegend	141702
31	168Er	CD39	Biolegend	135702
32	169Tm	CD69	Biolegend	104502
33	170Er	CD45R(B220)	Biolegend	103202
34	171Yb	CD86	Biolegend	105002
35	172Yb	CD127	Biolegend	135002

36	173Yb	CD196(CCR6)	Biolegend	129802
37	174Yb	CD192(CCR2)	RD	MAB55381-100
38	175Lu	MHC II(I-A/I-E)	Biolegend	107602
39	176Yb	TCR γ/δ	Biolegend	118140
40	197Au	CD4	Biolegend	100576
41	198Pt	CD8a	Biolegend	100746
42	209Bi	CD11b	Biolegend	101202
